# Seasonal dynamics and factors shaping microbiomes in freshwater finfish earthen aquaculture ponds in Bangladesh

**DOI:** 10.1186/s40793-025-00687-5

**Published:** 2025-03-31

**Authors:** Sanjit C. Debnath, Dominique L. Chaput, Jamie McMurtrie, Ashley G. Bell, Ben Temperton, Chadag V. Mohan, Md. M. Alam, Neaz A. Hasan, Mohammad M. Haque, David Bass, Charles R. Tyler

**Affiliations:** 1 Faculty of Health and Life Sciences, University of Exeter, Exeter, Devon, EX4 4QD UK; 2 Sustainable Aquaculture Futures, University of Exeter, Exeter, Devon, EX4 4QD UK; 3 WorldFish, Bayan Lepas, Penang, Malaysia; 4 Department of Fishery Resources Conservation and Management, Khulna Agricultural University, Khulna, Bangladesh; 5 Department of Fisheries and Marine Bioscience, Bangabandhu Sheikh Mujibur Rahman Science and Technology University, Gopalganj, Bangladesh; 6 Department of Aquaculture, Bangladesh Agricultural University, Mymensingh, 2200 Bangladesh; 7 Weymouth Laboratory, Centre for Environment, Fisheries and Aquaculture Science (Cefas), Weymouth, UK

**Keywords:** Water Microbiome, Pangasius, Nile tilapia, Season, Environmental parameters

## Abstract

**Background:**

The pondwater microbiome is believed to play a key role in fish health, including shaping mucosal surface microbiomes that help to protect against disease. How different physiochemical features relating to season, geographical locations, as well as crop species shape the pond water microbiome in the finfish aquaculture system, is not well established. Pangasius (*Pangasianodon hypophthalmus*) and tilapia (*Oreochromis niloticus*) are two of the most widely farmed fish species and disease is a major impediment to the expansion of their production. We applied 16S and 18S rRNA metabarcoding to assess how pond physicochemistry and geographical location shape water microbiomes in pangasius and tilapia aquaculture earthen ponds in Bangladesh.

**Results:**

Planctomycetota, Pseudomonadota and Actinomycetota were the dominant bacterial phyla while Stramenopiles and Alveolata were the dominant microeukaryotes (divisions) in the pangasius and tilapia ponds water. The relative abundance of Planctomycetota was higher in the pangasius ponds compared with tilapia ponds, and Actinomycetota, and Pseudomonadota were relatively higher in tilapia ponds. Tilapia pond water also exhibited a higher microbial diversity compared to that in pangasius ponds. The pondwater microbial diversity was at its lowest in winter (and/or in monsoon) and highest in the pre-monsoon period. The microbial community structures differed across the different seasons, geographical locations, culture systems, and crop species, with season and geographical locations showing the strongest effects. Of the water physicochemistry features assessed, temperature and pH were found to have a weak but significant effect on the water microbiome content for both pangasius and tilapia ponds. Pangasius and tilapia ponds shared over 46% of ASVs, and around 30% of ASVs were shared across the different study geographical locations.

**Conclusion:**

Our findings demonstrate that microbial communities in pangasius and tilapia aquaculture systems in Bangladesh are shaped by season, geographical location, crop species, as well as effects from water physicochemistry. Our results provide insights into the dynamic nature and environmental influences on water microbiomes that may be applied for use in pond management for improving aquaculture productivity and enhancement of overall fish health.

**Graphical Abstract:**

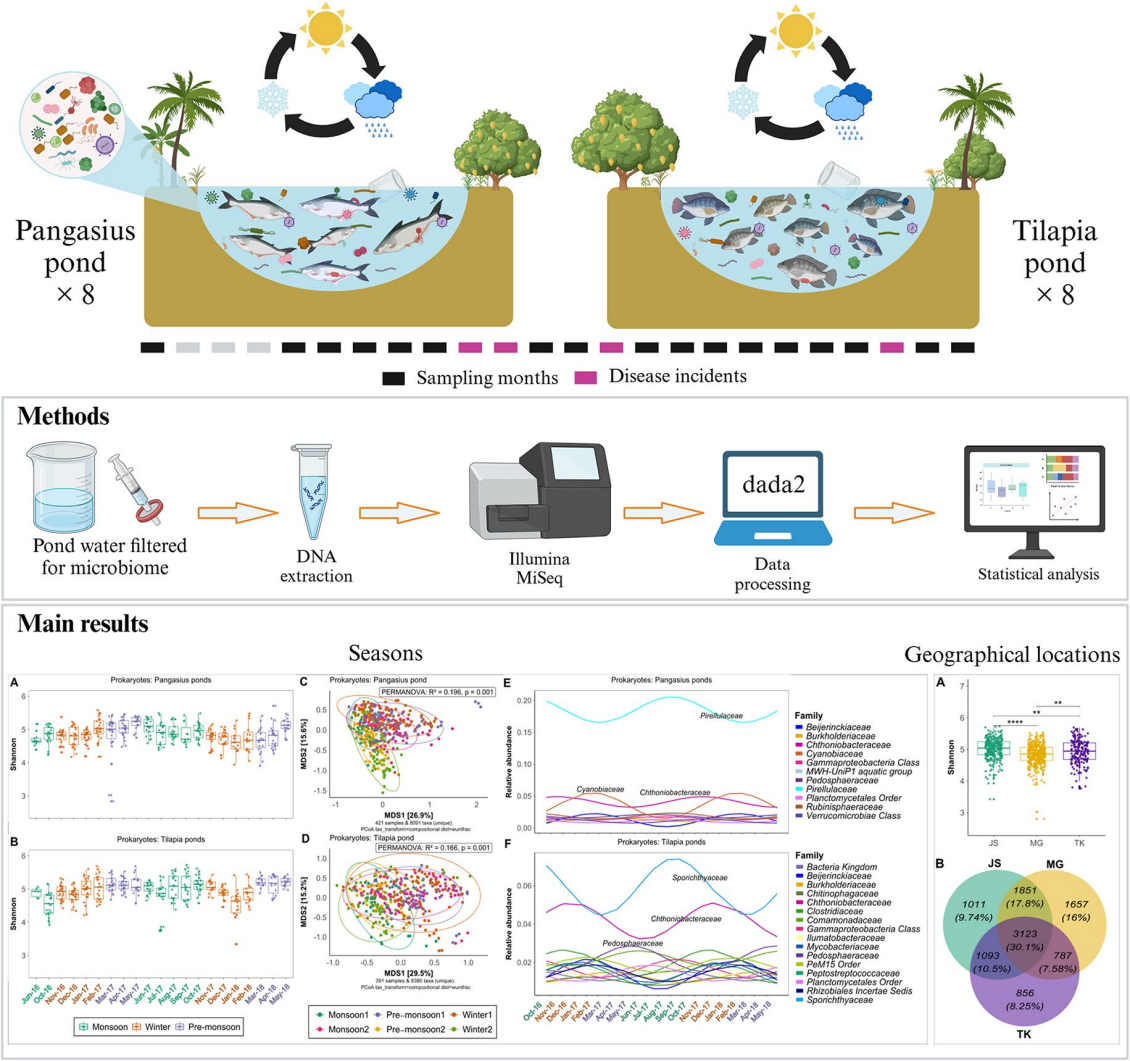

**Supplementary Information:**

The online version contains supplementary material available at 10.1186/s40793-025-00687-5.

## Introduction

The community of all living microorganisms that inhabit a body space are referred to as the microbiota, whilst the microbiome encompasses the microbiota and its associated functionality and downstream products [1]. Microbiomes are an essential part of fish and different body compartments differ in their microbiota [2–4]. Both exogenous (biotic and abiotic factors including the water habitat [5, 6], culture system [7–9], season [10, 11], geographical location [6, 12–14], and water physicochemistry [15]), as well as host-related endogenous factors, including host species [6] and infectious disease(s) [16] can influence the fish mucosal (gill, and/or skin, and/or gut) microbial composition and dynamics, either indirectly though the effect on the pond microbiome and/or directly to the fish [4, 17].

Microbiomes of the fish as well as the surrounding aquatic ecosystem play key roles in affecting overall fish health in aquaculture [18, 19]. Microbiomes of surrounding aquatic ecosystems play pivotal roles in maintaining water quality by enhancing productivity and facilitating various important nutrient cycles, such as nitrogen [20], carbon and phosphorus cycles [21], and removing toxic nitrogenous products from aquatic ecosystems by nitrification and denitrification [20], thus providing suitable environments for fish. Features of water physicochemistry, such as water temperature, salinity, pH, dissolved oxygen (DO), and nutrients influence the microbial structures and dynamics [22–24] and thus have a key role in shaping the water microbiome. The microbial community can respond to environmental change either by the microbes undergoing physiological adaptation and/or through compositional changes in the microbial community e.g. favouring the abundance of certain taxa more suited to the changing condition [25]. Fluctuations in the microbial structure of the aquatic ecosystem that may arise due to variations of the biotic and abiotic factors can profoundly impact fish health making them more, or less, susceptible to disease [17, 19, 26]. These influences act via various metabolites, peptides and proteins produced by the microbiota that in turn can interact with the host organism, affecting the host’s well-being by regulating various physiological functions [27]. In the case of water temperature, for Nile tilapia *Oreochromis niloticus* both high (over 35 °C) and low (below 22 °C) water temperatures can alter water microbiomes in ways that negatively impact fish growth [28, 29], elevate stress levels [30] and weaken their immune system, making them more susceptible to infection [31, 32]. Changes in water salinity have also been shown to result in shifts in the microbial community structure [25], which in turn can influence fish health, including through alterations to the fish’s microbiome. For instance, in Nile tilapia hypersaline conditions (24 ppt) can alter the gut microbiome, and increase opportunistic bacterial abundance while decreasing commensal/beneficial bacteria [33]. Similarly, pH [24, 34] and nutrient inputs from excess feed, organic matter, and fish excrements [35–37] have been shown to influence both the water and fish (skin and gut) microbial communities. Excessive growth of heterotrophic bacteria resulting from excess nutrient input that leads to hypoxia in the water body has been associated with the increase of pathogenic bacteria [38]. Thus, understanding the impact of the factors that shape the water microbiome can provide insights into how they influence the aquatic system and support fish health.

Aquaculture is hugely important for meeting the demand for nutritious and affordable food for billions of people globally. Currently, aquaculture contributes over 49% of aquatic products and around 17% of global animal protein, most of which are used for human consumption [39]. In Bangladesh, pangasius (*Pangasianodon hypophthalamus*) and tilapia *Oreochromis niloticus*/*O*. *mossambicus*) are the most farmed fish in inland pond aquaculture systems with an annual production of 395,615 tonnes and 329,316 tonnes, respectively [40]. Most of these fish species are consumed domestically [41–43] illustrating the importance of these fish species in the national diet of Bangladesh. However, with intensification, fish are becoming more susceptible to various infectious diseases which is a major constraint limiting global aquaculture production with an estimated loss of more than US$ 6 billion annually [44, 45]. Limited access to proper infrastructure, veterinary support and effective disease diagnostics is limiting the sustainable intensification of aquaculture in Bangladesh [43]. Moreover, limited vaccination and inadequate health management are failing to effectively address the disease problem in Bangladeshi aquaculture [46]. Understanding how different environmental factors shape the water microbiome and influence fish health is key to predicting possible disease outbreaks in aquaculture farms. There have been various studies on microbial community structures in aquaculture water systems [47, 48], however, very few studies have been conducted to explore the interrelationships between the microbial community dynamics in aquaculture, with season, culture systems, or geographical locations.

In this study, we aimed to investigate the interrelationships between the microbial assemblages in pangasius (*Pangasianodon hypophthalamus*) and tilapia (*Oreochromis niloticus*/*O*. *mossambicus*) aquaculture ponds and various environmental parameters. We hypothesised that the pondwater microbiome is shaped by multiple factors including pond temperature, salinity, pH, DO and geographical locations. We carried out this study in the Mymensingh division in Northern Bangladesh as this is one of the major areas in Bangladesh for inland fish production. Within this division, Jamalpur (covering Jamaplur Sadar upazila) and Mymensingh (covering Tarakanda and Muktagacha upazilas) districts produce 75,001-100,000 tonnes, and 250,000 tonnes of finfish, respectively [40]. The pond microbial prokaryotic and microeukaryotic compositions were analysed in 16 aquaculture ponds across three upazilas (subdistricts) at monthly intervals over a period of 21 months using 16S and 18S rRNA metabarcoding.

## Materials and methods

### Sampling overview

**Fig. 1:**
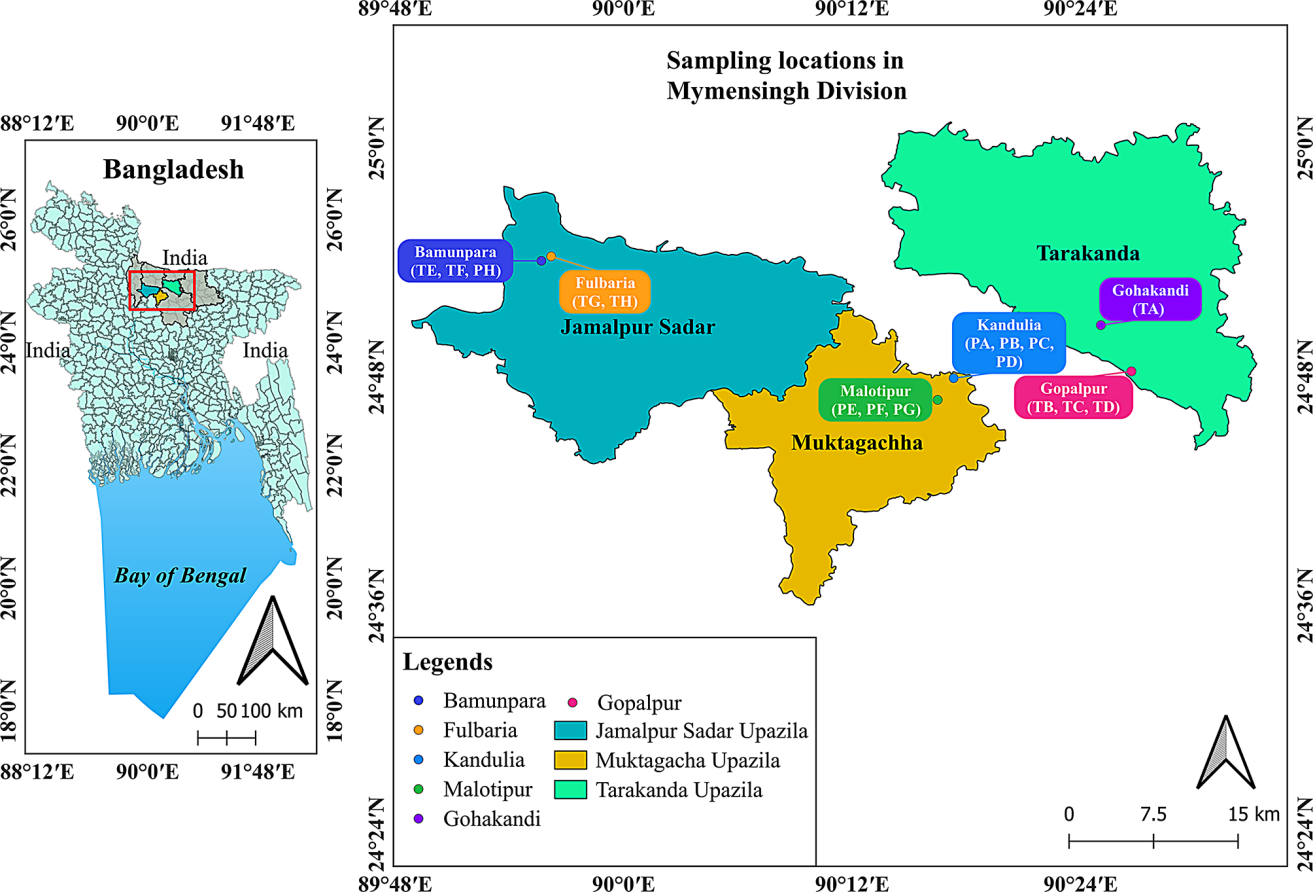
Locations of the pangasius and tilapia aquaculture ponds in Mymensingh Division, Bangladesh. Dots with different colours show villages from where samples were collected from different upazilas in the Mymensingh division. PA-PF: Pangasius ponds; TA-TF: Tilapia ponds

Metabarcoding was applied to investigate the microbial diversity of inland earthen aquaculture ponds in the Mymensingh division, Bangladesh. Sixteen fish farms were sampled across six different villages and three upazilas (subdistricts) over a period of 21 months (Fig. 1). Pangasius (*Pangasianodon hypophthalmus*) was the main crop in eight ponds (PA, PB, PC, PD, PE, PF, PG, PH) while Nile tilapia (*Oreochromis niloticus*) was the main crop in the other eight ponds (TA, TB, TC, TD, TE, TF, TG, TH) (Additional file 1, Table S1). Geographical distance from pond PA to others ranged from 110 m to more than 38 km (Fig. 1, Additional file 1, Table S1). From each pond, surface water samples were collected monthly (details provided below). After the eighth month of sampling (May 2017), one of the pangasius ponds (PH) switched the crop species from pangasius to a mix-culture of pangasius and tilapia and/or pangasius and carp. Across the tilapia farms, all ponds except ponds TA and TG switched from monoculture to polyculture after a certain period during our study period. All the fish farms were rural earthen ponds enclosed by earthen dykes. The size of the farms with pangasius ranged between around 1,550 m^2^ and 3,200 m^2^, and for tilapia, between around 1,200 m^2^ and 16,000 m^2^ (Additional file 1, Table S1). The depth of all these ponds varied between 1.1 m and 1.8 m. Both groundwater and rainwater were the main water sources. Depending on the available level of rainfall at different seasons, most of the ponds were generally topped up with ground water daily to weekly during the pre-monsoon period (March to May) and weekly to fortnightly during the other times of the year (June to October). In all ponds commercial floating/sinking feed was used during the study period.

### Sample collection for microbiome analysis

Monthly samples were collected from all 16 ponds between October 2016 and May 2018 (Additional file 1, Table S1). To collect microbial biomass, pond surface water was collected in triplicate (from three different locations) from each farm and passed through a polycarbonate membrane filter (47 mm diameter, 0.4 μm pore size, MerckMillipore, United States) using a 50 mL syringe. The filter was stored immediately in 100% molecular grade ethanol in a 2 mL cryogenic screw-cap tube. The volumes of filtered pond water ranged between 10 and 176 mL depending on the quantity of suspended particles in the pond water. Given the variable amount of suspended particles, it was not feasible to set a fixed sample volume; instead, pond water was passed through the filter until it clogged, resulting in a consistent DNA yield that was not correlated to the total volume of pond water (data not shown). A total of 919 and 920 samples were collected for 16S and 18S analysis, respectively across 16 farms during the 21-month study period (Additional file 1, Table S1). All the samples were stored at ambient temperature until arrival in the UK where they were stored at– 20 °C until processing for DNA extraction. In addition, water temperature (T), salinity, pH and dissolved oxygen (DO) were measured every month from March 2017 to May 2018 with an Extech DO610 ExStik II DO/pH/Conductivity Kit.

### DNA extraction and quantification

Ethanol was first removed by freeze-drying the samples at– 110 °C (ScanVac CoolSafe Pro; FRE4578, SLS) and thereafter, the filters were stored at– 80 °C. DNA extraction was performed through a CTAB/EDTA/chloroform method as previously described [14]. The full protocol is available at 10.17504/protocols.io.bw8gphtw. After extraction, DNA concentration was quantified using the Promega QuantiFluor ONE dsDNA quantification kit (E4870) with the Promega GloMax instrument, then normalised to 2ng/µL.

### PCR library preparation and sequencing

To amplify the small subunit ribosomal ribonucleic acid (SSU rRNA) marker genes of the prokaryotes and microeukaryotes from the collected samples, polymerase chain reaction (PCR) was performed with the Earth Microbiome Project recommended the V4 hypervariable region of the 16S rRNA gene (using 515 F (Parada) 5’-GTGYCAGCMGCCGCGGTAA-3′ [49] and 806R (Apprill) 5’-GGACTACNVGGGTWTCTAAT-3′ [50]); and the V9 hypervariable region of the 18S rRNA gene (using 1391f 5′-GTACACACCGCCCGTC-3′ [51] and EukBr 5′-TGATCCTTCTGCAGGTTCACCTAC-3′ [52]). The custom dual-indexing scheme and 1-step PCR approach of Kozich et al. [53] was adopted to allow batches of 384 barcoded samples to be multiplexed per sequencing run, with each batch including at least one mock community DNA standard and one extracted mock community standard (ZymoBIOMICS^®^), one DNA extraction blank, and one PCR blank. PCR thermocycling conditions for the 16S V4 region were as follows: an initial denaturation at 98 °C for 30s; followed by 30 cycles of denaturation at 98 °C for 10s, annealing at 55 °C for 30s, and extension at 72 °C for 30s; and a final extension of 72 °C for 2 min. For the 18S V9 amplification, all the conditions were the same except for the annealing temperature which was 60 °C. PCR reactions consisted of 25 µL NEBNext High-Fidelity PCR Master Mix (New England Biolabs), 17.5 µL nuclease-free water, 2.5 µL of 10 µM forward and reverse primers (final concentration 0.5 µM), and 2.5 µL of 2ng/µL DNA (final concentration 5 ng per reaction), in a total volume of 50 µL. Afterwards, PCR amplicons were visualised by 1.5% agarose gel electrophoresis. Amplicon libraries were quantified with Promega QuantiFluor ONE dsDNA quantification system (Promega, USA), pooled at an equimolar ratio, with the size and concentration of the final pool assessed using the TapeStation platform with High Sensitivity DNA ScreenTape (Agilent Technologies, United States). The pooled libraries were submitted to the University of Exeter Sequencing Service for sequencing on the Illumina MiSeq platform using v2 chemistry, 250 bp (300 bp for batch 1) paired-end for prokaryotic 16S rRNA and 150 bp paired-end for microeukaryotic 18S rRNA. Sequencing of the 16S samples was carried out over six sequencing batches whereas 18S samples were grouped into four batches. Triplicate samples from each pond and sampling date were spread across different sequencing batches to remove batch as a confounding variable.

### Bioinformatics and data analysis

#### Raw data processing and taxonomic assignment

After receiving the demultiplexed paired-end raw sequencing files, reads were processed using the DADA2 pipeline v1.26.0 [54]. In brief, read quality profiles for each sample for prokaryotes were checked and depending on an overall quality score over 30 for the forward and reverse reads, each sequencing batch was truncated at different positions (220 bp and 110 bp; 240 bp and 200 bp; 210 bp and 190 bp; 190 bp and 180 bp; 230 bp and 190 bp; 230 bp and 160 bp for sequencing batch B1, B2, B6, B7, B8 and B9, respectively). For the microeukaryotes, the forward and reverse reads were truncated at the 130 bp position. Amplicon sequence variants (ASVs) were subsequently inferred. To obtain the full denoised sequences, the forward and reverse reads were merged if both reads overlapped with a minimum of 45 bp and 30 bp for prokaryotes and microeukaryotes, respectively. Only merged reads of 250–256 bp for prokaryotes and 90–150 bp for microeukaryotes were kept to avoid off-target sequencing artefacts and chimaeras from each batch were identified and removed. After this, sequence tables from each batch were merged and used to construct the ASV table, and using the *assignTaxonomy* function from DADA2, taxonomy was assigned to prokaryotic ASVs using the SILVA SSU V138.1 taxonomic database [55] and to micoeukaryotic ASVs using the PR2 v5.0.0 taxonomic database [56]. To construct a phylogenetic tree of ASVs, sequences were aligned with MAFFT v7.475 [57] before determining the best-fitting model with ModelTest-NG v0.1.7 [58]. Model selection was made according to the lowest-scoring Akaike information criterion (AIC) and Bayesian information criterion (BIC) scores. The final phylogenetic tree was constructed with IQTREE v2.1.2 [59] using a GTR + I + G4 model. The tree was rooted by using the longest terminal branch as an outgroup.

All statistical analyses were performed in RStudio v2024.04.02.764 [60] using R v4.3.3 [61]. R package tidyverse v2.0.0 [62] for data manipulation, ggplot2 v3.5.1 [63], microViz v0.12.1 [64], cowplot v1.1.3, ggpubr v0.6.0 [65], VennDiagram v1.7.3 [66] and microeco v1.8.0 [67] packages for data visualisation were used. An ASV table (constructed with the merged sequenced table) representing amplicon sequence variants, a taxonomy table providing taxonomic assignments produced by the DADA2 pipeline, a phylogenetic tree elucidating evolutionary relationships among ASVs and a sample metadata table were amalgamated to construct a phyloseq object using the Phyloseq v1.46.0 package [68], which was later used for further quality control and downstream statistical analysis. Rigorous quality checks of the data were performed retaining ASVs that were present in > = 2% (prevalence threshold > = 2) for all samples. Likely microbial contaminants were identified using Decontam package v1.18.0 [69] with a prevalence threshold of 0.5 and these were removed before further analysis. Moreover, from the 16S data, any ASVs assigned as Chloroplast (rank = Order), Mitochondria (rank = Family), Eukaryota, Archaea, or unclassified (NA) at the Kingdom level were discarded and for microeukaryotes, any ASVs identified as Craniata (rank = Class), Teleostei (rank = Family), bacteria or unclassified (NA) at the Domain level were removed [54, 70]. Samples with less than 2000 read counts were also removed before further analysis. After the filtering, out of 919 and 920 samples sequenced for 16S and 18S respectively, 891 samples for 16S and 872 samples for 18S remained for the downstream analysis. Samples from ponds, where the culture system had been completely switched to a different fish species during the monitoring period (e.g. Shing, Gulsha and Carp, Gulsha and Pabda) were not included in the analysis.

#### Statistical analysis

Alpha diversity was estimated to characterise the within-sample microbial richness and evenness based on the rarefied data and the *estimate_richness* function within the Phyloseq package v1.42.0 [68] was used to compute the Shannon index [71], offering insights into community richness and evenness. Differences in alpha diversity across different sample groups (e.g. geographical locations (upazila), culture systems) were assessed using Kruskal–Wallis tests followed by pairwise comparisons using the post-hoc Dunn test with Benjamini-Hochberg (BH) adjustment to control for false discovery rate (FDR) for multiple comparisons.

To monitor changes in the prokaryotes and microeukaryotes community structure in the aquaculture pond across different seasons, geographical locations and culture systems, beta diversity was computed based on the phylogenetic distance on compositional transformed ASVs using weighted UniFrac distance metric [72] using microViz v0.12.10 [64] package. Ordination was performed with Principal coordinate analysis (PCoA) and visualised through ggplot2 [63]. A PERMANOVA (permutational multivariate statistical analysis of community separation) test [73] was carried out using the *adonis2* function of the vegan v2.6.6.1 package [74] to test for differences in beta diversity between groups mentioned above and *pairwise.adonis* function from the pairwiseAdonis v0.4.1 package was used for pairwise comparison [75] to check if the variance of taxonomic compositions differed when compared between different groups.

To assess the effect of seasonal variation on alpha diversity (Shannon index), a generalised linear model (GLM) with a Gamma distribution and a log link function was applied, using the model specified as glm (Shannon ∼ Season). After generating the Gamma and Negative Binomial model for both pangasius and tilapia pond, we used the Akaike Information Criterion (AIC) values calculated using *AIC*() function from base R, to select the best-fitting model. The gamma model had the lowest AIC, indicating the best fit, thus was chosen for this analysis. Pairwise comparisons between seasons were performed using estimated marginal means (EMMeans) using Emmeans v1.10.3 [76] R package, with the BH method to control FDR across multiple tests. Additionally, to identify taxa with seasonal trends (from October 2016 to May 2018) we applied a harmonic linear model following Bolaños et al. [77]. To mimic the seasonal cycle, we applied the function Xc = cos 2πt, to determine the day-length peaking in mid-winter, where t = d/365 being “d” the number of days since the winter solstice, and function Xs = sin 2πt to determine the day-length peaking at other times of the year where t = d/365 being “d” the number of days since 1st January. Taxa were aggregated at the family level and their relative contributions were tested to evaluate whether they followed the linear regression model. Additionally, *p*-values were adjusted for multiple regression tests using the BH method and families with *p*-value below 0.05 and mean relative abundance greater than 1% were plotted. Redundancy analysis (RDA) was performed to correlate environmental factors with microbial communities using the microViz packages. To explore the combined effect of seasons, geographical locations (Upazila), culture system, and water physicochemistry on the overall microbial community structure, we used a subset of samples for which all these variables were available and applied a PERMANOVA model with subsequent pairwiseAdonis test with BH method for multiple testing, as mentioned above. The formula used was adonis2(weighted UniFrac distance ~ Season + Upazila + Culture system + Salinity + Temperature + pH + DO, data = metadata).

## Results

### Water physicochemical features across the seasons

**Fig. 2:**
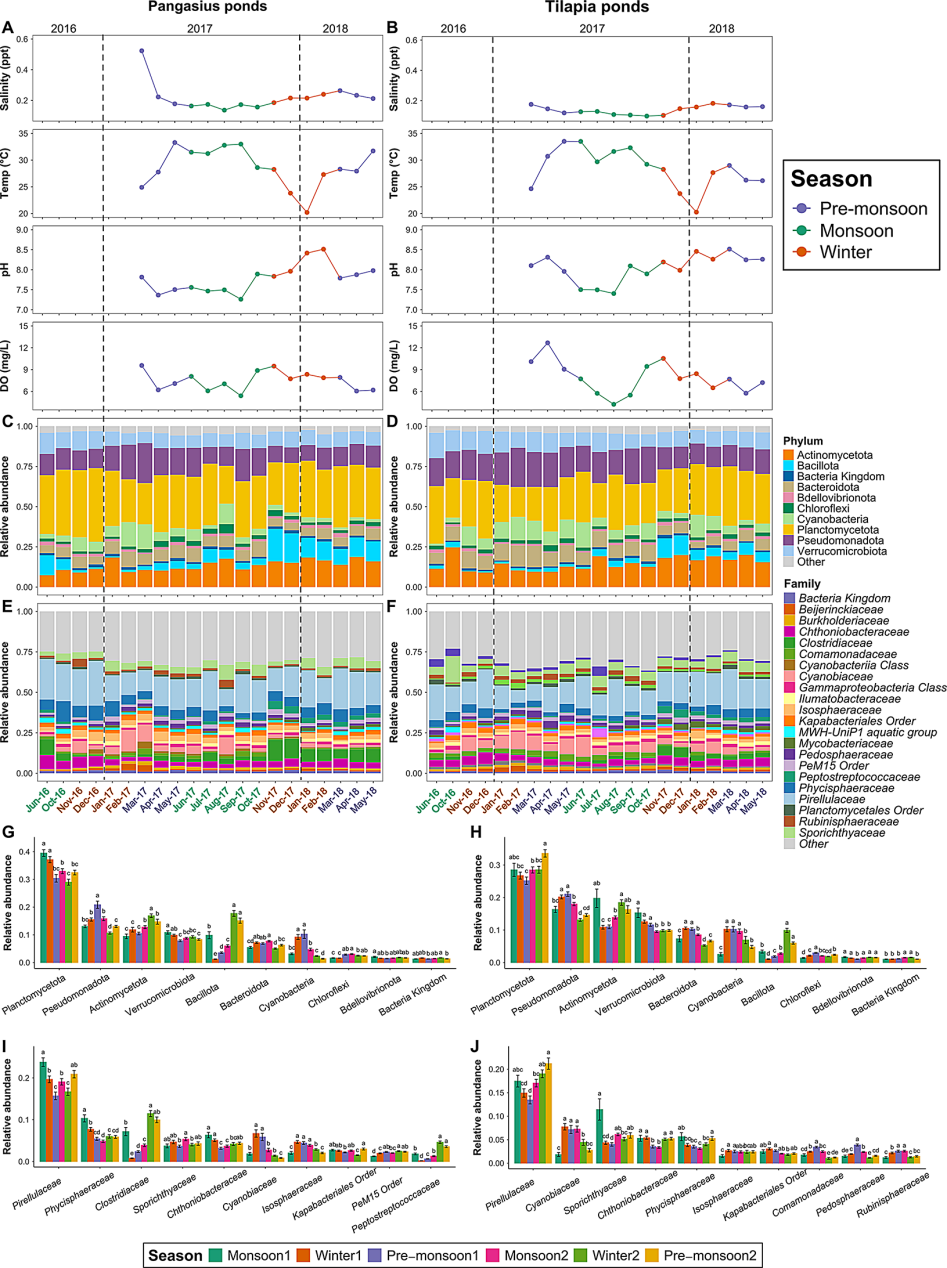
Seasonal dynamics of the water physiochemistry and microbial populations in pangasius and tilapia aquaculture ponds. Monthly salinity, temperature, pH, and DO profile in pangasius ponds (**A**) and tilapia ponds (**B**). There were no records for the physiochemical parameters for the first six sampling months. Pangasius pond water bacterial composition at the phylum level (**C**) and family level (**E**). Tilapia pond water bacterial composition at the phylum level (**D**) and family level (**F**). Composition and relative abundance are grouped based on the sampling month; each bar represents the mean relative abundance of the bacterial taxon within a group. Bacterial phyla and families with a mean relative abundance of ≥ 1% in overall samples are shown, the rest are combined as Others. The bar plots (**G**-**J**) display the seasonal variations in relative abundance for the top 10 taxa in pangasius and tilapia pond water. Statistical significances were assessed using the Kruskal-Wallis test, followed by Dunn’s post-hoc test for pairwise comparisons. Groups sharing the same letter(s) are not significantly different at *p*-adjusted < 0.05. Bar plots on the bottom left show the relative abundance of the top 10 bacterial phyla (**G**) and family (**I**) in pangasius ponds. The bar plots on the bottom right show the relative abundance of the top 10 bacterial phyla (**H**) and family (**J**) in tilapia ponds

Water samples collected from 16 ponds from three upazilas (subdistrict) over 21 months showed a wide range of variation in the recorded environmental parameters (Fig. 2A-B, Additional file 2, Table S2). Some variation was also seen in the water physicochemical parameters between the two pre-monsoon seasons (in 2017 and 2018). Notably, the water parameters showed a higher degree of fluctuations in pre-monsoon 2017 (further referred to as pre-monsoon1) compared to pre-monsoon 2018 (further referred to as pre-monsoon2) (Fig. 2A-B, Additional file 2, Table S2). For instance, in pangasius ponds, the temperature ranged between 24.89 and 33.29 °C in pre-monsoon1 while in pre-monsoon2, the temperature remained relatively stable, ranging only between 28.30 and 31.72 °C. Similar variability was also observed for other parameters in pangasius as well as in tilapia ponds (Additional file 2, Table S2). Local climate variability (e.g. rainfall patterns, temperature fluctuations) or pond management practices (e.g. planktonic growth) between the two years might be responsible for causing the interannual differences.

### Sequencing data profiles and seasonal water microbiome dynamics in pangasius and tilapia ponds

After all quality filtering procedures and removal of low-quality reads, we retained 10,358,751 high-quality sequence reads for prokaryotes (*n* = 812) and 7,676,770 for microeukaryotes (*n* = 797) datasets. This resulted in a mean read depth of 12,757 sequences per sample for prokaryotes and 9,632 for microeukaryotes. To assess whether the sampling depth was sufficient to give a representative overview of the pond water microbial communities, rarefaction curves for 16S and 18S sequences were generated. Both rarefaction curves reached a plateau, indicating that our sequencing effort was adequate to capture most of the microbial diversity present in each sample (see Additional file 3, Fig. S1). Details on the sequencing profiles are available in Additional file 4, Supplementary Table S3.

The bacterial communities in pangasius and tilapia pond water were dominated by phyla Planctomycetota (28 − 33%), Pseudomonadota (15 − 18%), Actinomycetota (13 − 14%) and Verrucomicrobiota (9 − 11%); and the family *Pirellulaceae* (17 − 19%) (Fig. 2C-F). However, the relative abundance of bacterial taxa varied significantly (Kruskal-Wallis, FDR < 0.05) between the different seasons (Fig. 2G-J). Of the most prevalent families, there was an increase in the abundance of the *Cyanobiaceae* family (belonging to the Cyanobacteria phylum) in both pangasius and tilapia ponds from the mid-winter season, in 2017 (further referred to as Winter1) (Fig. 2E-F, G-H). In addition, the relative abundance of the *Clostridiaceae* family belonging to Bacillota phylum was reduced significantly in winter1 compared with in monsoon1 and then increased again in the following winter (in pangasius; Fig. 2E, I). The dominant microeukaryotic taxa in both pangasius and tilapia ponds were divisions Stramenopiles (34 − 41%), Alveolata (17 − 24%), and Discoba (10 − 11%), and family *Stephanodiscaceae* (13 − 22%) (Additional file 3, Fig. S2). Similarly to that seen for the changes in bacteria, a seasonal change in microeukaryotes (albeit more subtle) was also observed in the ponds for both cultured fish species. Here, the relative abundance of the dominant family *Stephanodiscaceae* varied across different seasons in both pangasius and tilapia ponds (Additional file 3, Fig. S2G-H) and the *Euglenaceae* family varied through the different seasons in tilapia ponds (Additional file 3, Fig. S2H).

**Fig. 3:**
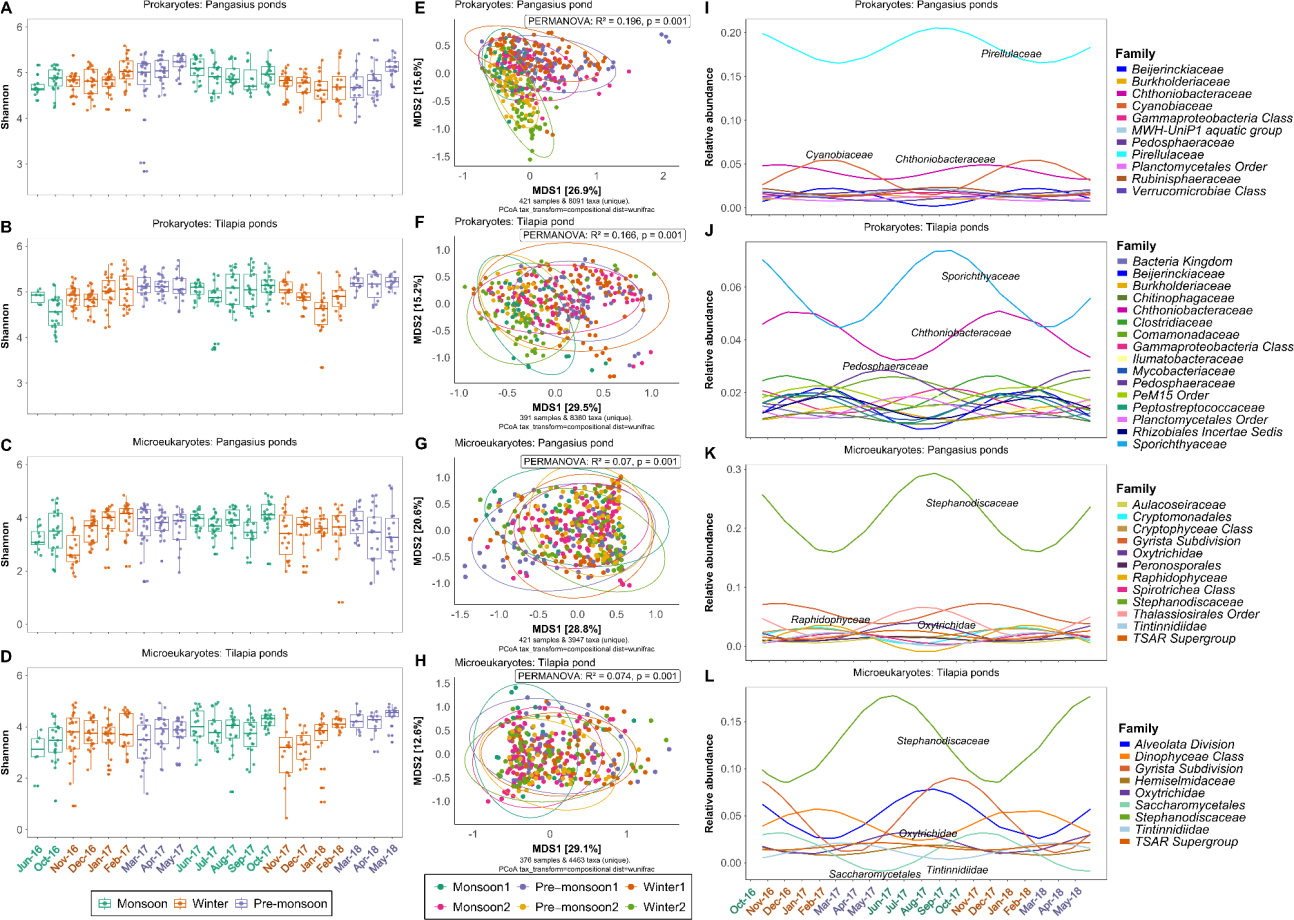
Microbial diversity and seasonal trends of microbial families in pangasius and tilapia ponds. The left panel shows bacterial alpha diversity (Shannon) in pangasius ponds (**A**), tilapia ponds (**B**), and microeukaryotic alpha diversity in pangasius ponds (**C**), and tilapia ponds (**D**). The PCoA plots in the middle show the beta diversity based on weighted-UniFrac distance matrix based on compositional data showing bacterial communities in pangasius ponds (**E**) and tilapia ponds (**E**), microeukaryotic communities in pangasius ponds (**G**) and tilapia ponds (**H**). The right panel shows the time-series of bacterial families in pangasius ponds (**I**), tilapia ponds (**J**), and microeukaryotic families in pangasius ponds (**K**), and tilapia ponds (**L**). A harmonic linear regression model was used to identify the significant seasonal trend (FDR < 0.05) of microbial families with a mean relative abundance > 1%. The first sampling month (June 2016) was excluded from the harmonic regression analysis due to a three-month gap before the next sampling period (October 2016). Sampling month names are coloured according to season

Analysis of alpha diversity (Shannon index) across different seasons for both pangasius and tilapia ponds clearly illustrated significant seasonal differences in microbial diversity (Fig. 3). For the prokaryotic communities in pangasius ponds, the microbial alpha diversity was higher in pre-monsoon1 (Mar-17 to May-17) and monsoon2 (Jun-17 to Oct-17) compared to winter2 (Nov-17 to Feb-18) (GLM, FDR < 0.05, Additional file 5, Table S4). There were no significant differences between other seasons (FDR > 0.05) in pangasius ponds. For microeukaryotic communities, the highest diversity occurred in monsoon2 and this was significantly greater than for the monsoon1 (Jun-16 to Oct-16), winter1 (Nov-16 to Feb-17), winter2 and pre-monsoon2 periods (Mar-18 to May-18) (GLM, FDR < 0.05; Additional file 5, Table S4). The seasonal changes for microeukaryotes in pangasius pond water were greater than that for the prokaryotes.

For tilapia, overall, microbial (alpha) diversity (both prokaryotic and microeukaryotic) showed more pronounced seasonal variation compared to that seen in pangasius ponds. Based on the estimated marginal means (log-transformed) from the GLM, a clear seasonal trend in bacterial diversity was observed in tilapia pond water, increasing from monsoon1 reaching a peak at pre-monsoon1, followed by a gradual decrease from monsoon2 with the lowest diversity in winter2 before increasing again (Additional file 6, Table S5). Pre-monsoon2 had the highest bacterial diversity (EMmean = 1.65), while monsoon1 had the lowest diversity (EMmean = 1.53). Pairwise comparisons confirmed that monsoon1 had significantly lower bacterial diversity compared to all other seasons in the tilapia pond water (GLM, FDR < 0.01, Additional file 6, Table S5). In addition, pre-monsoon1 had significantly higher diversity compared with winter1 and winter2 (GLM, FDR < 0.001). The diversity increased in pre-monsoon2 compared to winter2 (GLM, FDR < 0.0001, Additional file 6, Table S5), suggesting an overall trend of increasing prokaryotic diversity from the monsoon through to the pre-monsoon seasons in the tilapia pond water. For microeukaryotes, based on the estimated marginal means, a seasonal trend of diversity was also observed, with pre-monsoon2 having the highest alpha diversity (EMmean = 1.45), and monsoon1 the lowest diversity (EMmean = 1.21, Additional file 6, Table S5) in the tilapia pond water. Pairwise comparisons further illustrated significant differences between seasons; compared with monsoon1, diversity in winter1, monsoon2, pre-monsoon2 was increased (GLM, FDR < 0.05), diversity in pre-monsoon2 was greater compared to winter1 (GLM, FDR < 0.001), diversity in both monsoon2 and pre-monsoon2 was greater than winter2 (GLM, FDR < 0.001). These results suggest a trend for a decrease of microeukaryotic diversity from monsoon reaching the lowest level in winter and then increasing to the pre-monsoon period in the tilapia pond water.

Analysis of the beta diversity based on the weighted UniFrac distance matrix on compositional data showed that season strongly influences microbiome structure in pangasius and tilapia ponds. For the pangasius ponds, PERMANOVA analysis (to test if the microbiome composition differed between groups) revealed that season explained over 19% variation in the bacterial community structure (PERMANOVA: R^2^ = 0.196, *p* = 0.001) and 7% in microeukaryotes (Fig. 3E, G). Similarly, for tilapia ponds, over 16% variation in bacterial community structure and 7% for microeukaryotic communities were explained by seasons (Fig. 3F, H). However, the lack of any clear visual separation on the PCoA plots indicates that there is nevertheless a high degree of similarity in microbial composition across all seasons. Therefore, other factors are likely to make up most of the variation in microbiome composition in these pond ecosystems.

To further explore the seasonal trend of the microbial taxa, a harmonic regression model was performed. This modelling revealed that in pangasius ponds 139 bacterial families (out of 575) and 156 bacterial families (out of 566) in tilapia ponds showed significant seasonal trends (FDR < 0.05). Of these, 11 families in the pangasius pond and 16 families in the tilapia ponds had a mean relative abundance greater than 1% (Fig. 3I-J). The harmonic regression modelling revealed two consistent seasonal patterns in the microbial communities in both pangasius and tilapia ponds, with distinct peaks during the monsoon and winter periods (Fig. 3I-L). In pangasius ponds, for the prokaryotic communities, the relative abundance of *Pirellulaceae* and *Chthoniobacteraceae* families reached its peak during the monsoon period, while the *Cyanobiaceae* family dominating peak occurred in the winter (Fig. 3I). In contrast, the microbial communities in the tilapia ponds showed a slightly different seasonal pattern. There was a dominating *Sporichthyaceae* family in the monsoon period and the *Chthoniobacteraceae* family in the winter. In addition, there was a third peak emerging during the pre-monsoon period dominated by the *Pedosphaeraceae* family (Fig. 3J).

For microeukaryotes, in pangasius ponds, 75 families (out of 292) showed a significant seasonal trend among which 12 had a mean relative abundance greater than 1% (Fig. 3K). Of these, *Stephanodiscaceae* and *Oxytrichidae* families dominated during the monsoon, whereas in winter, taxa belonging to *Raphidophyceae*, *Cryptomonadales*, and *Tintinnidiidae* showed higher relative abundance (Fig. 3K). For tilapia, 69 microeukaryotic families (out of 305) showed a significant seasonal trend with nine having a mean relative abundance greater than 1% (Fig. 3L). Among these, the relative abundance of *Stephanodiscaceae* and *Oxytrichidae* families were higher in the monsoon, whereas *Saccharomycetales* showed a winter peak (Fig. 3L). Similar to bacterial communities, there was a third peak of microeukaryotic *Tintinnidiidae* showing a peak during the pre-monsoon period in tilapia (Fig. 3L). Thus, although many microbial taxa in both tilapia and pangasius ponds showed clear seasonal trends across the seasons with their relative abundance peaking in one specific season and reducing in another, the specific families contributing to these peaks differed between the two pond ecosystems. The results from these seasonal pattern analyses suggest that the microbial communities in each aquaculture system differ, being shaped by different environmental and/or biological factors, such as fish culture species.

### Effect of physicochemical factors on pond microbiomes

**Fig. 4:**
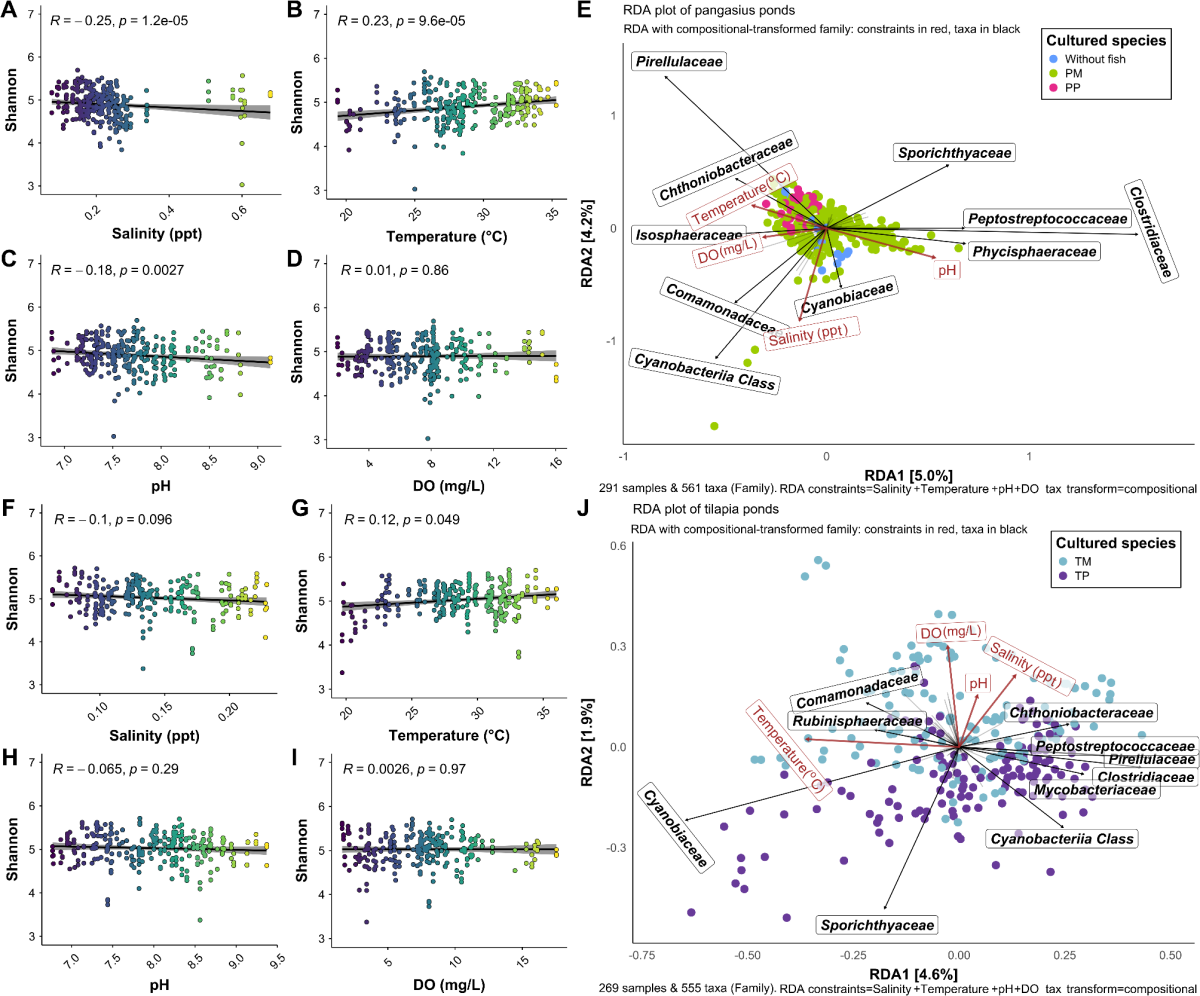
Spearman’s correlation and redundancy analysis of pangasius and tilapia pond water. Correlation analyses were carried out between bacterial alpha diversity (Shannon) and salinity, temperature, pH and DO using Spearman’s correlation (linear regression model) for pangasius ponds (**A**-**D**) and tilapia ponds (**F**-**I**). Redundancy analysis (RDA) presented shows the correlation among samples (fish species), environmental factors and bacterial families in Pangasius pond (**E**) and Tilapia pond (**J**). Black arrows show vectors of the significant microbes at family levels while red arrow shows vectors of the environmental factors. A positive correlation is indicated when the angle between vectors is less than 90°, while a negative correlation is indicated when it is greater than 90°. Any vector perpendicular to each other indicated no correlation

In pangasius ponds, the bacterial alpha diversity (Shannon) was positively correlated with temperature (*R* = 0.23, *p* < 0.0001) and negatively correlated with salinity (*R* =– 0.25, *p* < 0.0001) and pH (*R* =– 0.18, *p* < 0.01) (Fig. 4A-D). However, there was no significant correlation between alpha diversity and DO (*R* =– 0.007, *p* > 0.05) in pangasius ponds (Fig. 4A-D). For microeukaryotes in pangasius ponds, alpha diversity was negatively correlated with salinity (*R* =– 0.14, *p* < 0.05), but there were no significant correlations between alpha diversity for microeukaryotes with temperature, pH or DO (Additional file 3, Fig.S3A-D). In tilapia ponds, bacterial alpha diversity (Shannon) was positively correlated with temperature (*R* = 0.12, *p* = 0.049) but there were no significant correlations with DO (*R* = 0.0026, *p* > 0.05), salinity (*R* =– 0.1, *p* > 0.05), or pH (*R* =– 0.065, *p* > 0.05) (Fig. 4F-I). For microeukaryote communities, there were no significant correlations between the alpha diversity and salinity, temperature, or pH were observed, but there were for DO (*R* =– 0.19, *p* < 0.01) (Additional file 3, Fig. S3F-I).

To investigate for associations between specific microbial taxa and features of water physicochemistry, RDA analyses were performed. In pangasius ponds, *Pirellulaceae* and *Chthoniobacteraceae* were positively correlated with temperature and DO; *Isosphaeraceae* and *Comamonadaceae* were positively correlated with temperature, DO and salinity; *Cyanobiaceae* was positively correlated with pH and salinity; while *Sporichthyaceae*, *Peptostreptococcaceae*, *Clostridiaceae*, and *Phycisphaeraceae* were positively correlated with pH (Fig. 4E). In tilapia ponds, *Sporichthyaceae* and *Cyanobiaceae* were positively correlated with temperature; *Rubinisphaeraceae* and *Comamonadaceae* were positively correlated with temperature and DO; *Chthoniobacteraceae* was positively correlated with all the environmental factors, except temperature; *Peptostreptococcaceae*, *Clostridiaceae*, *Mycobacteriaceae*, and *Pirellulaceae* were positively correlated with pH and salinity (Fig. 4J).

For the microeukaryotes, in pangasius ponds, *Euglenaceae*, *Phacaceae*, and *Raphidophyceae* families were positively correlated with salinity; *Raphidophyceae* family was positively correlated with DO and pH; and *Oxytrichidae* and *Stephanodiscaceae* families were positively correlated with temperature (Additional file 3, Fig. S3E). In tilapia ponds, *Stephanodiscaceae* and *Chlamydomonadales* were positively correlated with temperature; and *Chlamydomonadales*, *Cryptomonadales*, and *Raphidophyceae* families were positively correlated with DO, pH and salinity (Additional file 3, Fig. S3J).

### Effect of culture system and crop species

**Fig. 5:**
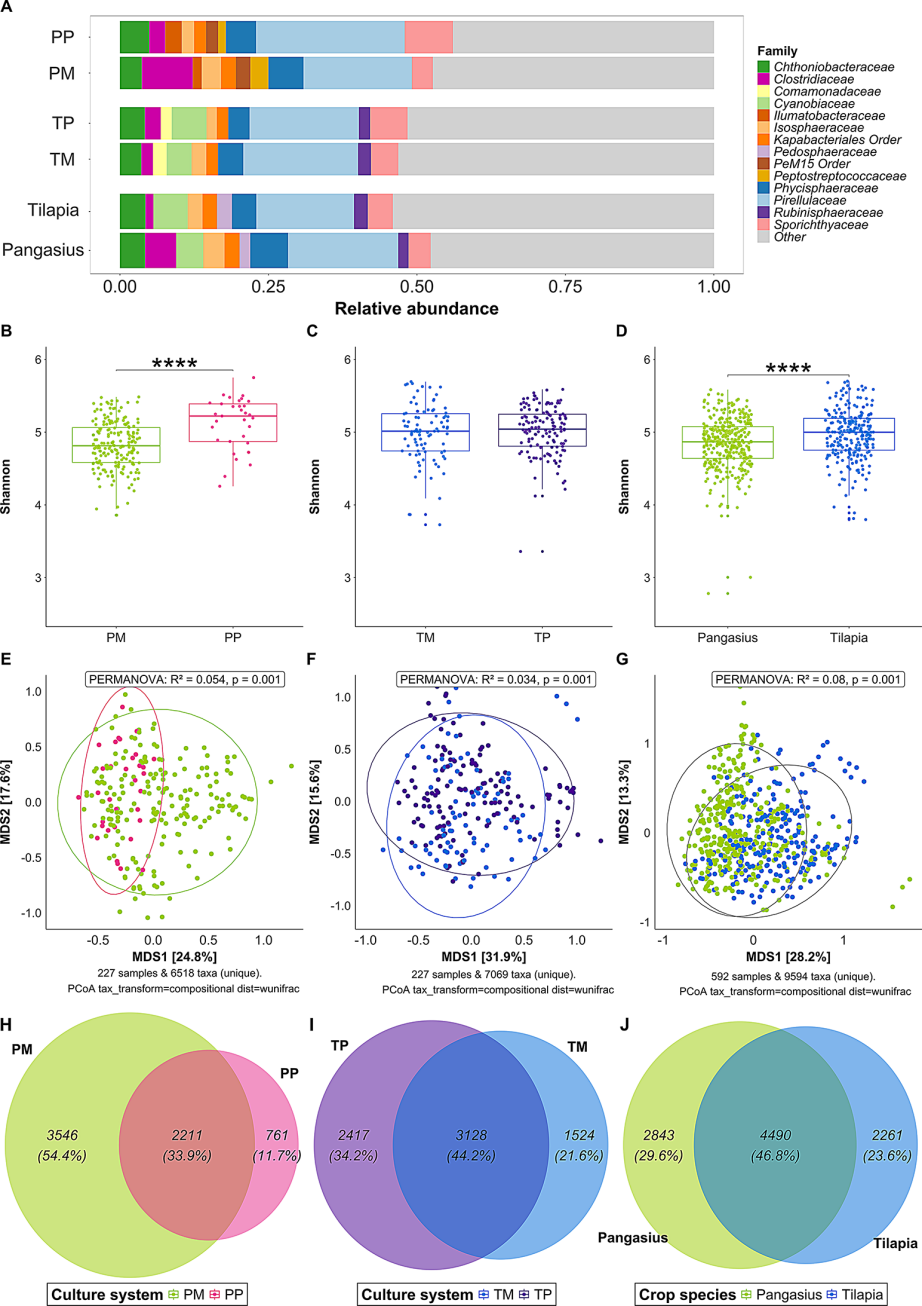
Bacterial composition, diversity and shared ASVs in fish mono- and polyculture systems and crop species. **A**) Bacterial composition of top 10 families in PM (Pangasius-monoculture), PP (Pangasius-polyculture), TM (Tilapia-monoculture), TP (Tilapia-polyculture), pangasius and tilapia (ponds with only pangasius and only tilapia) culture systems. Box plots showing alpha diversity (Shannon) of pangasius-monoculture and polyculture system (**B**); tilapia-monoculture and polyculture system (**C**); pangasius and tilapia ponds (**D**). PCoA ordination plots with weighted-UniFrac distance matrix showing beta diversity between PM and PP systems (**E**); between TM and TP systems (**F**); and between pangasius and tilapia culture systems (**G**). Venn diagrams showing shared and unique ASVs between PM and PP (**H**), TM and TP (**I**), and pangasius-tilapia (**J**). Asterisks indicate statistically significant differences between groups using the Kruskal-Wallis test (*: *p*-value < 0.05; **: *p*-value < 0.01; ***: *p*-value < 0.001; ****: *p*-value < 0.0001)

In the ninth month of the field monitoring study (May 2017), pangasius ponds PH, and tilapia ponds TB, TC, TD, TF and TH were switched from single fish species cultures (i.e. pangasius or tilapia) to multiple finfish species (Additional file 1, Table S1) and we investigated how the microbiomes in these culture systems (for multiple species) differed compared with those seen in the pangasius and tilapia monoculture ponds. Mono- and polyculture systems (for both pangasius and tilapia ponds) had similar microbial community compositions, but they differed in the proportional representation at different taxonomic levels. In pangasius ponds, Planctomycetota (31 − 36%), Actinomycetota (14 − 16%), Pseudomonadota (13 − 14%), Bacillota (5 − 13%), and Verrucomicrobiota (8 − 11%) were the dominant phyla in the mono- and polyculture systems (Additional file 7, Table S6). Of the dominant phyla, the relative abundance of Planctomycetota, Actinomycetota, and Verrucomicrobiota was significantly higher (Kruskal-Wallis, *p* < 0.05) in pangasius-polyculture compared to pangasius-monoculture, while Bacillota was significantly higher (Kruskal-Wallis, *p* < 0.0001) in pangasius-monoculture compared to pangasius-polyculture (Additional file 7, Table S6). At the family level, pangasius mono- and polyculture systems were dominated by *Pirellulaceae*, *Clostridiaceae*, *Phycisphaeraceae* and *Sporichthyaceae* (Fig. 5A, Additional file 7, Table S6). The relative abundance of *Pirellulaceae* and *Sporichthyaceae* was significantly higher in pangasius-polyculture compared to pangasius-monoculture while *Clostridiaceae* and *Phycisphaeraceae* were significantly higher in pangasius-monoculture compared to pangasius-polyculture (Kruskal-Wallis, *p*-value < 0.001).

In tilapia ponds, Planctomycetota (27.66 − 31.63%%), Actinomycetota (12.83 − 17.50%), Pseudomonadota (15.52 − 16.93%), and Verrucomicrobiota (8.99 − 10.68%) were dominant in tilapia-monoculture and tilapia polyculture systems (Additional file 7, Table S6). The relative abundances of Planctomycetota, Pseudomonadota, and Verrucomicrobiota were significantly higher in tilapia-monoculture while Actinomycetota was significantly higher in tilapia-polyculture (Kruskal-Wallis, *p* < 0.05). At the family level, tilapia-monoculture and tilapia-polyculture systems were dominated by *Pirellulaceae*, *Sporichthyaceae* and *Cyanobiaceae* (Fig. 5A, Additional file 7, Table S6). Of these, the relative abundance of *Sporichthyaceae* was significantly higher in tilapia-polyculture compared to tilapia-monoculture (Kruskal-Wallis, *p*-value < 0.0001), with no significant differences for other dominant families.

To assess how the crop species affected the pondwater microbial composition, we compared ponds where only pangasius or only tilapia during the study period. Within pangasius and tilapia monoculture systems, Planctomycetota (29.39 − 33.51%), Pseudomonadota (15.32 − 19.11%), Actinomycetota (11.72 − 12.37%), and Verrucomicrobiota (9.19 − 12.30%) were the major phyla (Additional file 7, Table S6). Of these, Planctomycetota was significantly higher in pangasius ponds compared with tilapia ponds, while Actinomycetota and Pseudomonadota were significantly higher in tilapia ponds compared to pangasius ponds (Kruskal-Wallis, *p* < 0.05). At the family level, *Pirellulaceae*, *Phycisphaeraceae* and *Cyanobiaceae* families were dominant in both pangasius and tilapia ponds (Fig. 5A, Additional file 7, Table S6), but the relative abundance of *Pirellulaceae* and *Phycisphaeraceae* was significantly higher in pangasius ponds compared to tilapia ponds (Kruskal-Wallis, *p*-value < 0.01), whilst *Cyanobiaceae* was significantly higher in tilapia ponds compared to pangasius ponds (Kruskal-Wallis, *p*-value < 0.0001).

Microbial alpha diversity was significantly different (Kruskal-Wallis, *p*-value < 0.0001) between pangasius-monoculture and pangasius-polyculture (Fig. 5B), while for tilapia, this was not the case, with no significant difference observed between the tilapia-monoculture and tilapia-polyculture ponds (Fig. 5C). The major crop species (pangasius and tilapia) however did influence the pond microbiome, with significant differences (Kruskal-Wallis, *p*-value < 0.0001) between them (Fig. 5D). Beta diversity analysis using PCoA based on weighted-UniFrac dissimilarities showed a considerable overlap of microbial communities for all groups, indicating a considerable proportion of shared taxa between the different culture groups (Fig. 5E-G). PERMANOVA findings, however, indicated weak, but significant differences in microbial communities between pangasius-monoculture and pangasius-polyculture (PERMANOVA: R^2^ = 0.05, *p* = 0.001), between tilapia-monoculture and tilapia-polyculture (PERMANOVA: R^2^ = 0.03, *p* = 0.001) and between pangasius and tilapia (PERMANOVA: R^2^ = 0.08, *p* = 0.001) (Fig. 5E-G), illustrating different culture system and different crop species exert an influence over the structure of pond water microbial communities. Assessing for unique and shared prokaryotes ASVs between these different groups, we found that pangasius-monoculture and pangasius-polyculture shared 33.9% (2211/6518) (Fig. 5H), while tilapia-monoculture and tilapia-polyculture shared over 44.2% (3128/7069) (Fig. 5I). Moreover, 46.8% (4490/9594) ASVs were found to be common in pangasius and tilapia ponds (Fig. 5J). Similar results were seen for the microeukaryotic community composition and diversity (Fig. S4).

### Effect of geographical location

A higher microbial diversity was found in water samples collected from pangasius ponds in the Jamalpur Sadar upazila compared with the Muktagacha upazila (Kruskal-Wallis, *p*-value < 0.0001, Additional file 3, Fig. S5A). For the microeukaryotic communities, there was no significant difference (*p* > 0.05) in the diversity (alpha and beta) between these two locations (Additional file 3, Fig. S5B). For the tilapia pond samples collected from the Jamalpur Sadar and Tarakanda upazilas, the microbial alpha diversity for both prokaryotes and microeukaryotes differed significantly (Kruskal-Wallis, *p*-value < 0.05) (Additional file 3, Fig. S5C-D). In terms of beta diversity, for both pangasius (PERMANOVA: R^2^ = 0.02, *p* = 0.001) and tilapia (PERMANOVA: R^2^ = 0.07, *p* = 0.001 for prokaryotes, PERMANOVA: R^2^ = 0.05, *p* = 0.001 for microeukaryotes) ponds microbial diversity differed, but only weakly (Additional file 3, Fig. S5A, C, D).

**Fig. 6:**
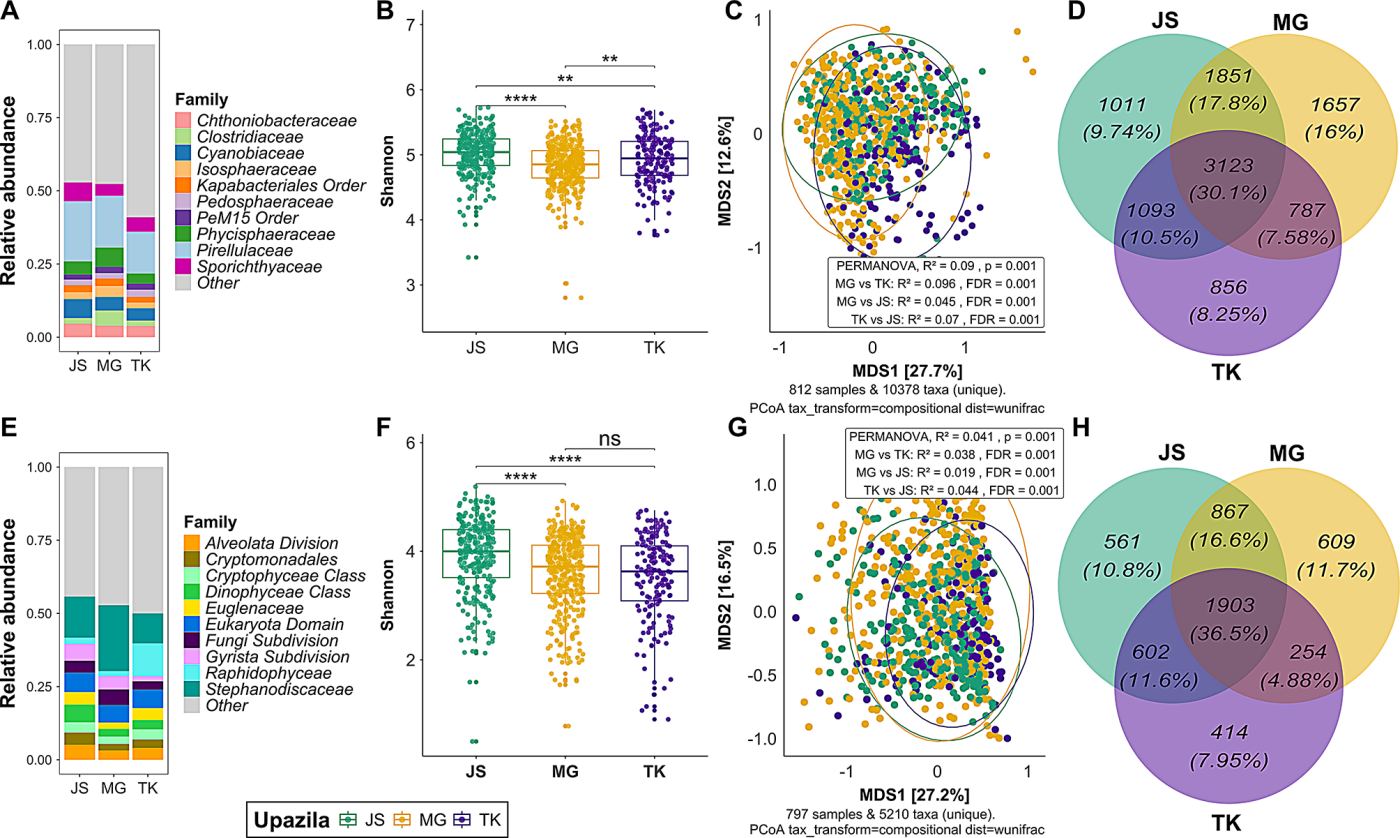
Comparison of microbial composition, diversity and shared ASVs across the different studied geographical locations. The upper panel shows the top 10 bacterial family composition (**A**), Shannon diversity (**B**), beta diversity based on weighted-UniFrac distance (**C**) and shared ASVs (**D**) from different upazilas. The bottom panel shows family-level microeukaryotic composition (**E**), Shannon diversity (**F**), beta diversity based on weighted-UniFrac distance (**G**) and shared ASVs (**H**) from different upazila. JS: Jamalpur Sadar, MG: Muktagacha, TK: Tarakanda. Asterisks indicate statistically significant differences in pairwise comparisons with the Kruskal-Wallis test (ns = not-significant, *: FDR < 0.05; **: FDR < 0.01; ***: FDR < 0.001; ****: FDR < 0.0001)

When combining the pangasius and tilapia pond data, the effect of geographical location on the microbiome communities was more pronounced. Microbial composition in all three upazilas was overall similar but with different proportional representations of the different taxonomic ranks. At the family level, *Pirellulaceae* (14 − 20%), *Cyanobiaceae* (3 − 7%), and *Sporichthyaceae* (4 − 6%) were dominant in overall all upazilas and were significantly different (Kruskal-Wallis, FDR < 0.05) between three upazilas (Fig. 6A, Additional file 8, Table S7). Alpha diversity (Shannon) comparison between groups indicated that the bacterial communities differed significantly (Kruskal-Wallis, FDR < 0.01) between all upazilas (Fig. 6B), although a PCoA plot showed a clear overlap of the microbial communities between three upazilas. PERMANOVA followed by the pairwise comparison indicated that there was a weak but significant association between upazila and pond water microbiome composition (Fig. 6C). Overall, more than 30% of ASVs were shared between ponds across the different upazilas (Fig. 6D).

At the family level, most of the microeukaryotic reads could not be classified. Among the classified families, *Stephanodiscaceae* was the most dominant (Fig. 6E), and this differed significantly across all three upazilas (Kruskal-Wallis, FDR < 0.05). In addition, microeukaryotic alpha diversity in Jamalpur Sadar was greater than in the Muktagacha and Tarakanda upazilas (Kruskal-Wallis, FDR < 0.0001, Fig. 6F). There was a significant, albeit weak influence of geographical location on beta diversity of the pond water microbiome composition (Fig. 6G). There was a relatively higher (36.5%) shared microeukaryote community between the three upazilas compared with that seen for bacterial communities (Fig. 6H).

### The overall effect of all studied variables

PERMANOVA followed by a pairwise-adonis test applied to assess the combined influence of seasons, geographical locations (upazila), culture system, and water physicochemistry (temperature, salinity, pH and DO) on the overall microbial (prokaryotes) community structure (for samples from June-2017 to May-2018) revealed a significant influence (*p*-value < 0.05) of all these variables on the overall microbial community structure (Table 1). Of these factors, seasons (PERMANOVA: R^2^ = 0.12, *p*-value = 0.001) and geographic locations (PERMANOVA: R^2^ = 0.13, *p*-value = 0.001) were the best predictors indicating the highest effect on microbial community compositions (Table 1). PERMANOVA indicated a significant but weaker influence of the culture system (mono-polyculture). However, the pairwise comparisons revealed a more pronounced influence of crop species (R^2^ = 0.10, *p*-value = 0.001), suggesting in fact that crop species play a role in shaping the microbial community composition.

**Table 1:** Overall effect of different environmental factors on microbial (prokaryotes) structure. PERMANOVA and pairwise-adonis results on weighted-UniFrac distance matrix showing the effect of seasons, geographical locations (Upazila), culture system, and water physicochemistry on bacterial communities of the aquaculture pond water. Samples between June-2017 and May-2018 meet the criteria where all the aforementioned variables were available and therefore only these samples were included in this model. Significance code: *: *p*-value < 0.05; **: *p*-value ≤ 0.01; ***: *p*-value ≤ 0.001

Factor	R^2^	F	*p*-value	Significance
Season	0.12	37.67	0.001	***
Upazila	0.13	39.10	0.001	***
Culture system	0.04	11.39	0.001	***
Temperature	0.02	12.00	0.001	***
Salinity	0.01	4.80	0.002	**
pH	0.02	13.12	0.001	***
DO	0.01	4.91	0.001	***
**Pairwise comparison**	**R** ^**2**^	**F-Model**	***p*** **-adjusted (BH) value**	**Significance**
Monsoon2 vs. Winter2	0.12	42.54	0.001	***
Monsoon2 vs. Pre-monsoon2	0.09	26.58	0.001	***
Winter2 vs. Pre-monsoon2	0.04	10.32	0.001	***
Muktagacha vs. Jamalpur Sadar	0.11	41.68	0.001	***
Muktagacha vs. Tarakanda	0.13	35.97	0.001	***
Jamalpur Sadar vs. Tarakanda	0.07	17.31	0.001	***
Pangasius-monoculture vs. Pangasius-polyculture	0.06	12.81	0.001	***
Pangasius-monoculture vs. Tilapia-polyculture	0.12	42.04	0.001	***
Pangasius-polyculture vs. Tilapia-monoculture	0.07	7.99	0.001	***
Pangasius-polyculture vs. Tilapia-polyculture	0.07	11.29	0.001	***
Tilapia-monoculture vs. Tilapia-polyculture	0.03	6.54	0.001	***
Pangasius-monoculture vs. Tilapia-monoculture#	0.10	28.65	0.001	***

^#^These are also considered crop species as these were only pangasius and only tilapia

## Discussion

Maximising the growth and health of organisms in aquaculture can be enhanced through understanding the microbial compositions in culture systems, and in turn, optimising them to maintain good water quality and buffer against disease-causing pathogens. Various studies have reported that changes in microbial composition (dysbiosis) in the surrounding environment are associated with disease states in fish and other aquatic organisms [36, 78, 79], however, microbial populations are context-dependent, illustrating the needs to understand the specifics of any given system for optimising the microbiomes for supporting healthy culture environments.

In both the pangasius and tilapia pond waters, the most dominant (> 10%) bacterial phyla were found to belong to Planctomycetota, Pseudomonadota, and Actinomycetota, and this is consistent with that identified previously for tilapia and pangasius aquaculture ponds in Asia and also in Africa [14, 26, 48, 80–82]. Other studies characterising the microbial communities of the freshwater ponds (and lakes) more widely in Bangladesh have found a high prevalence also of Cyanobacteria and Bacteroidota [26, 83, 84]. In terms of microeukaryotes, the dominance of the family *Stephanodiscaceae* (and genus *Cyclotella*) in pangasius and tilapia pond waters is also consistent with that reported by McMurtrie et al. [14]. This differs, however, from that reported by Zhou et al. [26] where Opisthokonta, SAR, and Cryptophyceae were found to be the dominant taxa in tilapia ponds. Although these differences may, in part, relate to the regional geochemistry, nutrient content, and physicochemistry of the waters (see later), differences in the age of fish in the ponds may also be a contributing factor as the life stage in tilapia has been reported to influence microbial composition and diversity in culture ponds [26]. Both pangasius and tilapia pond microbiomes showed significant differences in both alpha and beta diversity between the geographical locations (upazilas). These findings of the pond microbial compositions across different upazilas align with previous work also [12, 14, 82, 84]. The fact that, of the sequenced microbial ASVs, only 30% were shared between the different upazilas, emphasises the importance of geographically local environmental factors shaping the water microbiota in fish farming ponds.

The Pseudomonadota, Planctomycetota, and Actinomycetota play key roles in promoting suitable water quality and thus are of considerable ecological significance for promoting good fish health in these aquaculture pond systems. Pseudomonadota are known to have fundamental roles in various biochemical processes including nitrogen, sulphur and carbon cycling [85] and in degrading dissolved organic matter [86]. Members of the Planctomycetota too play key roles in carbon and nitrogen cycles [87] and the Actinomycetota play crucial roles in degrading organic matter [85, 88] as well as producing various enzymes, immune modifiers, bioactive compounds belonging to macrolide and quinolone groups, and a range of natural peptide products [89]. Members of the Planctomycetota have also been identified to harbour antibiotic resistance genes (ARGs) with 14 out of 108 species in this phylum known to be resistant to various antimicrobials [90], including the *Pirellulaceae* family that was dominant in both pangasius and tilapia pond water in our study. Furthermore, recent studies have identified a wide range of ARGs conferring resistance to different classes of antimicrobials in these same aquaculture ponds [82] as well as in other pangasius and tilapia aquaculture farms across various districts (including Mymensingh) in Bangladesh [91].

The changes in the relative abundance and composition of microbial taxa with season highlight the highly dynamic nature of the microbiomes in these pond systems. Some bacteria, such as the *Pirellulaceae* family and the microeukarytic *Stephanodiscaceae* family were persistently dominant in the pond waters throughout all seasons. Members of the *Pirellulaceae* family are widely available in aquatic ecosystems playing key roles in nutrient cycles and utilise organic compounds [88, 92], while diatom *Stephanodiscaceae* is known as a primary producer [93], playing a crucial role in the aquatic food web. Therefore, the persistence of these taxa across all seasons suggests that these taxa might be able to thrive in a broad range of environmental conditions and might be less sensitive to seasonal temperature fluctuations. However, a wide variation of bacterial families such as *Phycisphaeraceae*, *Clostridiaceae*, and *Cyanobiaceae* in pangasius pond, and both bacterial (*Cyanobiaceae*) and microeukaryotic (*Sporichthyaceae* and *Euglenaceae*) families in tilapia ponds across the different seasons were also observed in the present study. Some of these seasonal fluctuations in abundance appear to be temperature related with a higher microbial diversity in the warmer months of the pre-monsoon period and lowest during the winter months. The RDA and harmonic regression analysis we performed show a clear association between specific microbial families and temperature. These results concur with previous studies seen for shifts in gut microbial communities in Atlantic salmon (*Salmo salar*) [94], Chinook salmon (*Oncorhynchus tshawytscha*) [95] and rainbow trout (*Oncorhynchus mykiss*) [96] depending on the variations in water temperature and seasonality. Temperature plays an important role in fish growth and metabolism, and increasing water temperatures would affect (increase) their excretion rates and fish-derived nutrients in water [97]. Many members of the *Cyanobiaceae* family, for instance, are sensitive to temperature, causing bloom during warmer months often above 25 °C [98], benefiting from high nutrients, increased sunlight and temperature [99]. Generally, Cyanobacteria is not toxic but some species (such as *Microcystis* spp. and *Planktothrix* spp.) can produce toxic [98], which is harmful to the environment, organisms (fish) living in the water and to human. Thus, temperature and nutrient-flux across different seasons might be responsible for influencing specific taxa in the aquatic ecosystem. Moreover, temperature extremes also tend to support the growth of disease-causing microbial taxa in aquaculture systems [31, 100]. In our study, the mean temperature of both pangasius and tilapia ponds were within their optimal range for growth (for pangasius this is between 28 and 32 °C [28, 30, 101] and for tilapia between 26 and 30 °C [29, 30]) and there were no major disease outbreaks reported throughout the study in the ponds sampled, which highlights the importance of maintaining optimum temperature in aquaculture system.

Acidic conditions can invoke acid stress [102] with notable changes in the fish microbiomes [24, 34]. A decrease in water microbial alpha diversity occurred in pangasius ponds with increasing pH, consistent with findings from a previous study on pangasius in Bangladesh [103]. Interestingly, there was no such relationship seen between pH and microbial alpha diversity in the tilapia ponds. Our RDA analysis revealed specific bacterial families, such as *Cyanobiaceae*, *Sporichthyaceae*, *Peptostreptococcaceae*, *Clostridiaceae*, and *Phycisphaeraceae* were positively correlated with the pH variation in the tilapia culture ponds. Although some species of the family *Clostridiaceae* such as *Clostridium difficile* is a well-known pathogen, this was not abundant in our study and also no obvious disease outbreak was observed during the study. Tilapia have been shown to have a wide pH tolerance range (between 5.5 and 9.0) [102, 104–106] and the pond water pH in our study was well within this range. Therefore, this pH range may not have been extreme enough to promote the proliferation of pathogenic microorganisms in the studied ponds. Moreover, the positive correlation of the above-mentioned specific families to pH is most likely that these taxa were present as part of the natural microbiomes which are primarily associated with primary production, nutrient cycling and ecosystem functioning. Recognising the importance of maintaining water pH for fish health, applying lime to do so is commonly practised in Bangladeshi aquaculture farms [107].

The finding for the effects of water salinity on pond microbiomes also differed between the pangasius and tilapia ponds. There was a decrease in water microbial alpha diversity (Shannon) with increasing salinity in pangasius ponds, but there was no such significant correlation in tilapia ponds. This finding likely relates that the salinity range in tilapia ponds (0.099–0.182 ppt (‰)) was considerably less than that occurring in the pangasius ponds (0.137–0.523‰). In terms of dissolved oxygen (DO) level, generally, tilapia and pangasius can tolerate low DO, for tilapia even down to 0.01 mg/L [30] and as low as 0.05–0.1 mg/L for pangasius [108], due to their ability to extract oxygen through gulping air [109, 110]. The measured DO level in both pangasius and tilapia ponds in this study ranged between 4 and 13 mg/L and this overall good level of pond oxygenation likely explains the lack of any major effect on bacterial diversity (Shannon) for either of the fish species culture ponds. It is known that the diurnal patterns of DO can vary widely, especially under conditions of algal blooms, and this will likely affect the microbial assemblages in those ponds. However, little has been researched in this regard for pangasius and tilapia culture systems.

In our study, although the dominant phyla in both pond systems were Planctomycetota, Pseudomonadota and Actinomycetota, there were substantial differences in the microbial composition (mean relative abundance), and diversity (in both alpha and beta) between the ponds culturing pangasius or tilapia. Thus, highlighting fish species can influence the water microbiome in these earthen pond systems. In a study of tilapia and pangasius ponds across five geographical regions in Bangladesh by Islam et al. [103], total coliforms and *Escherichia coli* were also found to differ between pangasius and tilapia ponds (they were relatively higher in pangasius farms). In another study on the microbial compositions of pond waters, fish species (that included tilapia) were also reported to influence the pond water microbiota, albeit modestly so [80]. The gut microbiome communities (and skin microbiomes) can show high levels of variation between different fish species [2, 6, 111], even when the different fish are maintained in the same habitat [112–114]. It is thus likely that the distinct gut microbiomes of pangasius and tilapia, through excretion, help to account for the different pond microbial environments seen. Interestingly, of the top phyla recorded in our study, Cyanobacteria was significantly higher in tilapia ponds compared to pangasius ponds. Although it is not known why this occurred, this may be due to differences in pond management practices between the two species (e.g. differences in food provision), the influence of the fish species and /or other factors. Nile tilapia is an omnivorous filter-feeding fish and consumes significant amounts of Cyanobacteria [115]. As such their enhancement in these pond systems may contribute positively to their production, but equally excess Cyanobacteria may have detrimental impacts on fish health.

Evidence from both factor-specific and multivariate PERMANOVA analyses showed higher microbial diversity in pangasius-polyculture systems compared with pangasius-monoculture systems. This was not the case for the microbial alpha diversity in the tilapia ponds, which may suggest that the tilapia has a greater influence on the overall alpha diversity. Nevertheless, the microbial structure and assemblages did differ for tilapia between the monoculture and polyculture systems. These findings suggest that different fish species have different degrees of influence on the overall pond microbiome diversity and that the microbial communities in the tilapia ponds may be more stable, or rather less easily influenced by other extraneous factors, than for the case of pangasius. This could be influenced by pond management practices, types of feed provided, and differences in feeding behaviours between different fish species.

## Conclusion

Applying 16S and 18S rRNA metabarcoding we illustrate the pond microbiome diversity varies with crop species (pangasius versus tilapia), culture systems (monoculture and polyculture), and geographical locations across three different upazilas of Bangladesh, and is also well-marked by seasonality. We identify a series of common microbiota in pangasius and tilapia pond waters across all geographical regions regardless of the physiochemical variation in both pond systems across the studied regions. Furthermore, we show that the different culture systems influence unique microbial profiles with strong correlations between pondwater microbial communities and water physicochemistry, illustrating the importance of water conditions in fostering a favourable microbial community in aquaculture systems. The basic characterisations of these microbiomes in different fish culture systems and how various seasonality, physiochemical and environmental factors shape the dynamic nature of those microbiomes provide the basis for developing microbial diagnostic features for disease and other harmful conditions for managing and developing enhanced productivity in pond-based aquaculture systems across different upazilas in Bangladesh.

## Electronic supplementary material

Below is the link to the electronic supplementary material.

Supplementary Material 1: Additional file 1, Table S1. Details on water samples collected from Pangasius and Tilapia aquaculture ponds in Mymensingh, Bangladesh. Light green and teal colours denote ponds with only pangasius and tilapia, respectively. Light blue colour indicates mixed culture and orange colour shows where the main crop switched to other fish species. Numbers in parentheses denote samples for 18 S analysis.

Supplementary Material 2: Additional file 2, Table S2. Seasonal variations in water physicochemical features in the aquaculture ponds. Values are presented as the mean (± SD) of monthly samples across all ponds. Water physicochemical parameters data were not collected before March 2017.

Supplementary Material 3: Additional file 3. Supplementary figures (Figure caption provided for each figure).

Supplementary Material 4: Additional file 4. Table S3. Sequencing profiles of the prokaryotes (16 S) and microeukaryotes (18 S) datasets. Data presented following the different types of filtering and sample removal where the reads were less than 2000.

Supplementary Material 5: Additional file 5. Table S4. Seasonal variation in the microbial communities in pangasius ponds based on a generalised linear model. Winter1: Jun-Oct, 2016, pre-monsoon1: Nov 2016-Feb 2017, monsoon1: Mar-May 2017, winter2: Jun-Oct 2017, pre-monsoon2: Nov 2017-Feb 2018, monsoon2: Nov 2016-Feb 2018.

Supplementary Material 6: Additional file 6. Table S5. Seasonal variation in the microbial communities in tilapia ponds based on a generalised linear model. Winter1: Jun-Oct, 2016, pre-monsoon1: Nov 2016-Feb 2017, monsoon1: Mar-May 2017, winter2: Jun-Oct 2017, pre-monsoon2: Nov 2017-Feb 2018, monsoon2: Nov 2016-Feb 2018.

Supplementary Material 7: Additional file 7. Table S6. Mean relative abundance and pairwise comparison of bacterial phyla and families in different culture systems. Mean relative abundance (Mean RA), Standard deviation (SD), and Standard error of the mean (SEM) were calculated for the top 10 phyla and families for pangasius monoculture and polyculture systems; tilapia monoculture and polyculture system; and for pangasius and tilapia (only for those ponds where single species was maintained). Kruskal-Wallis tests were performed using the relative abundance of each phylum/family to compare the difference between groups, with relative abundance values calculated for each phylum/family within each sample. ns = not-significant, *: *p*-value < 0.05; **: *p*-value < 0.01; ***: *p*-value < 0.001; ****: *p*-value < 0.0001.

Supplementary Material 8: Additional file 8. Table S7. Mean relative abundance and pairwise comparison of bacterial families across the different geographical locations. Mean relative abundance (Mean RA), Standard deviation (SD), and Standard error of the mean (SEM) were calculated at different upazila for pangasius and tilapia. Kruskal-Wallis test followed by the post hoc Dunn test was performed using the relative abundance of each family to compare the difference between groups, with relative abundance values calculated for each family within each sample. ns = not-significant, *: *p*-value < 0.05; **: *p*-value < 0.01; ***: *p*-value < 0.001; ****: *p*-value < 0.0001.

## Data Availability

The 16 S and 18 S sequencing raw data were deposited in the European Nucleotide Archive (ENA) under BioProject accession number PRJEB82362 and are publically available. Data processing and analysis scripts are available at https://github.com/DebnathSanjit/Mymensingh_pond_water_microbiomes. The final ASV tables and phyloseq objects will be made publicly available upon publication of this work or on request.

## Abbreviations

16S rRNA: 16 S ribosomal ribonucleic acid
18S rRNA: 18 S ribosomal ribonucleic acid
AIC: Akaike information criterion
ASV: Amplicon sequence variants
BH: Benjamini-Hochberg
DNA: Deoxyribonucleic acid
DO: Dissolved oxygen
EMMeans: Estimated marginal means
FDR: False discovery rate
GLM: Generalised linear model
PCoA: Principal coordinate analysis
PCR: Polymerase chain reaction
PERMANOVA: Permutational multivariate statistical analysis of variance
PM: Pangasius monoculture
PP: Pangasius polyculture
ppt: parts per thousand
RDA: Redundancy analysis
TM: Tilapia monoculture
TP: Tilapia polyculture
