# Supplementary material for: Seasonal dynamics and factors shaping microbiomes in freshwater finfish earthen aquaculture ponds in Bangladesh
Source: Environ Microbiome. 2025 Mar 31;20:38. doi: 10.1186/s40793-025-00687-5 (PMC11960027; doi:10.1186/s40793-025-00687-5)
Supplement: Supplementary file 3 — Supplementary Material 3: Additional file 3. Supplementary figures (Figure caption provided for each figure). [file 40793_2025_687_MOESM3_ESM.docx]

**Seasonal dynamics and factors shaping microbiomes in freshwater finfish earthen aquaculture ponds in Bangladesh**

Sanjit C. Debnath^1,2,†^, Dominique L. Chaput^1,2^, Jamie McMurtrie^1,2^, Ashley G. Bell^1,2^, Ben Temperton^1^, Chadag V. Mohan^3^, Md. M. Alam^4^, Neaz A. Hasan^5^, Mohammad M. Haque^6^, David Bass^2,7^, Charles R. Tyler^1,2,†^

1 Faculty of Health and Life Sciences, University of Exeter, Exeter EX4 4QD, Devon, UK

2 Sustainable Aquaculture Futures, University of Exeter, Exeter EX4 4QD, Devon, UK

3 WorldFish, Bayan Lepas, Penang, Malaysia

4 Department of Fishery Resources Conservation and Management, Khulna Agricultural University, Khulna, Bangladesh

5 Department of Fisheries and Marine Bioscience, Bangabandhu Sheikh Mujibur Rahman Science and Technology University, Gopalganj, Bangladesh

6 Department of Aquaculture, Bangladesh Agricultural University, Mymensingh 2200, Bangladesh

7 Weymouth Laboratory, Centre for Environment, Fisheries and Aquaculture Science (Cefas), Weymouth, UK

**†Correspondence**

scd226@exeter.ac.uk +44-(0)-1392-7654131

c.r.tyler@exeter.ac.uk +44-(0)-1392-7654131

**Submitted to:** *Environmental Microbiome*

### Supplementary figures


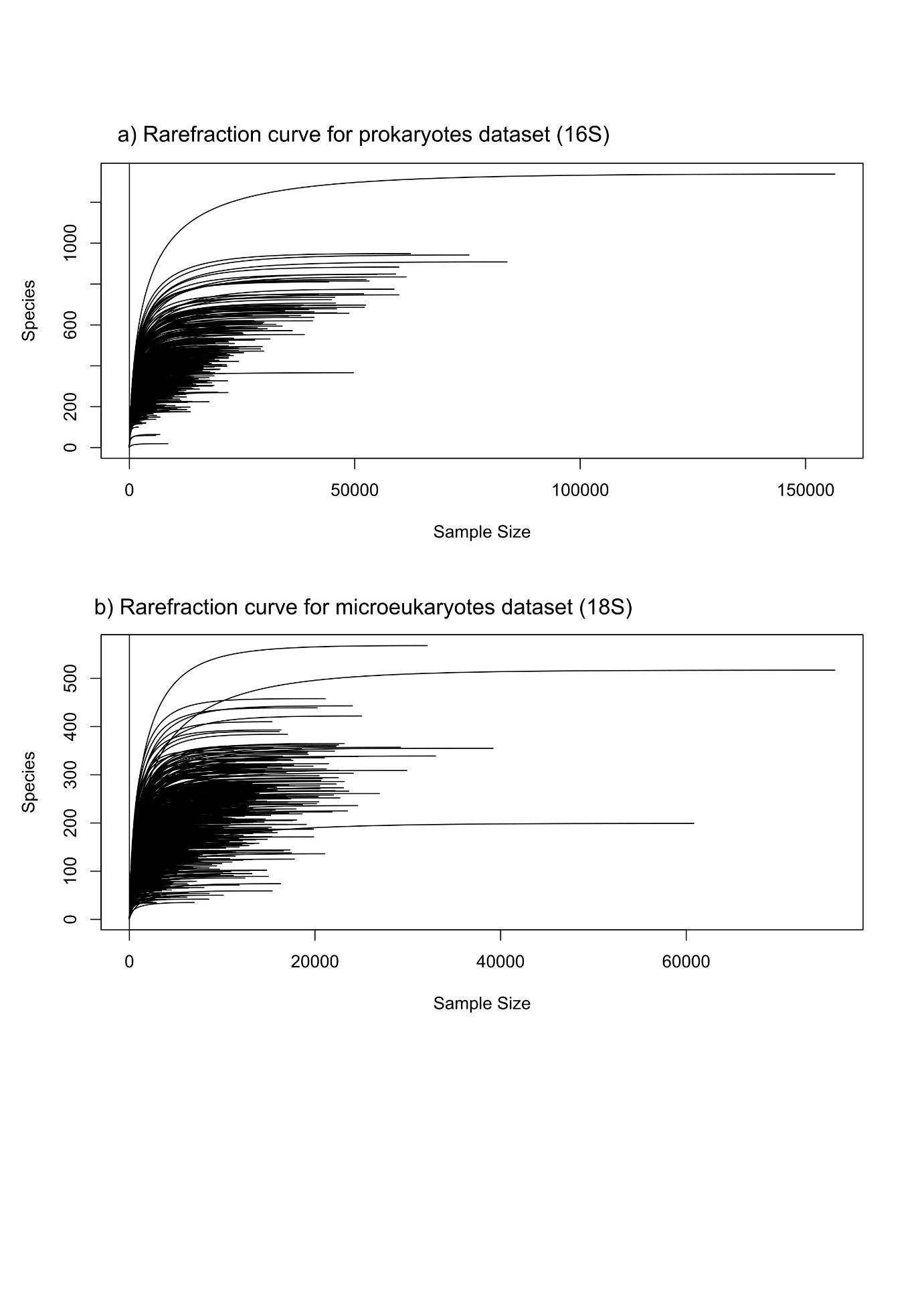


Fig. S1 Rarefaction curves of the prokaryotes and microeukaryotes communities based on observed ASVs.

a) Prokaryotic dataset, b) Microeukaryotic dataset.


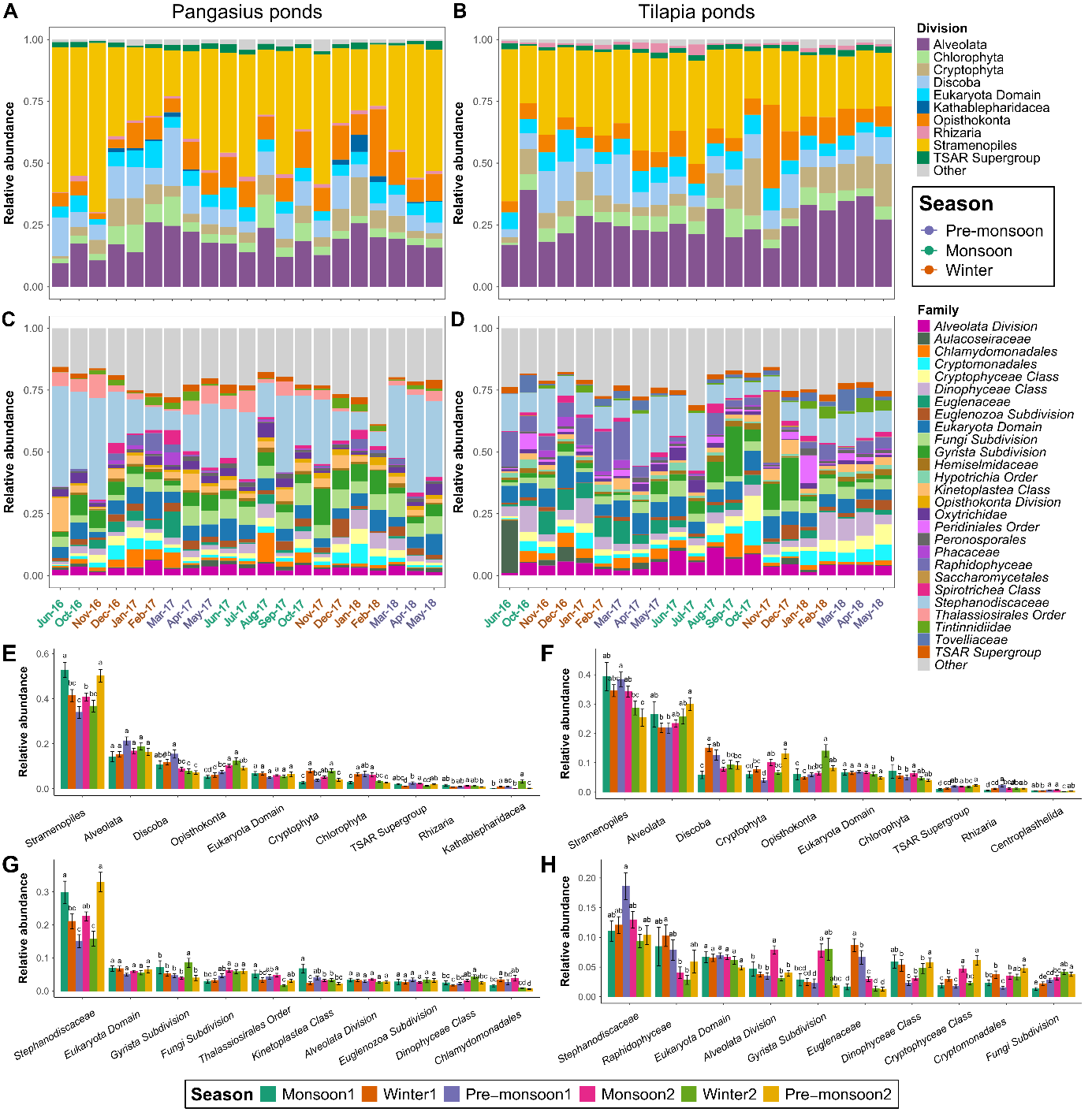


Fig. S2 Relative abundance of microeukaryotic divisions and families in pangasius and tilapia pond water.

A) Pangasius pond water microeukarytic composition at the division level. B) Tilapia pond water microeukarytic composition at the division level. C) Pangasius pond water microeukarytic composition at the family level. D) Tilapia pond water microeukarytic composition at the family level. Composition and relative abundance are grouped based on the sampling month; each bar represents the mean relative abundance of the microeukaryotic taxon within a given group. Microeukaryotic taxonomic Division and families with a mean relative abundance of ≥ 1% in overall samples are shown, the rest are combined as Others. At both the division and family level, taxa with higher rank (e.g. Eukaryota Domain, Alveolata Division) are those ASVs which cannot be classified into the desired (division/family) ranks. The bar plots (E-H) display the seasonal variations in relative abundance for the top 10 taxa in pangasius and tilapia pond water. Statistical significances were assessed using the Kruskal-Wallis test, followed by Dunn’s post-hoc test for pairwise comparisons. Groups sharing the same letter(s) are not significantly different at *p*-adjusted < 0.05. Bar plots on the bottom left show the relative abundance of the top 10 microeukaryotic divisions (E) and family (G) in pangasius ponds. The bar plots on the bottom right show the relative abundance of the top 10 microeukaryotic divisions (F) and family (H) in tilapia ponds.


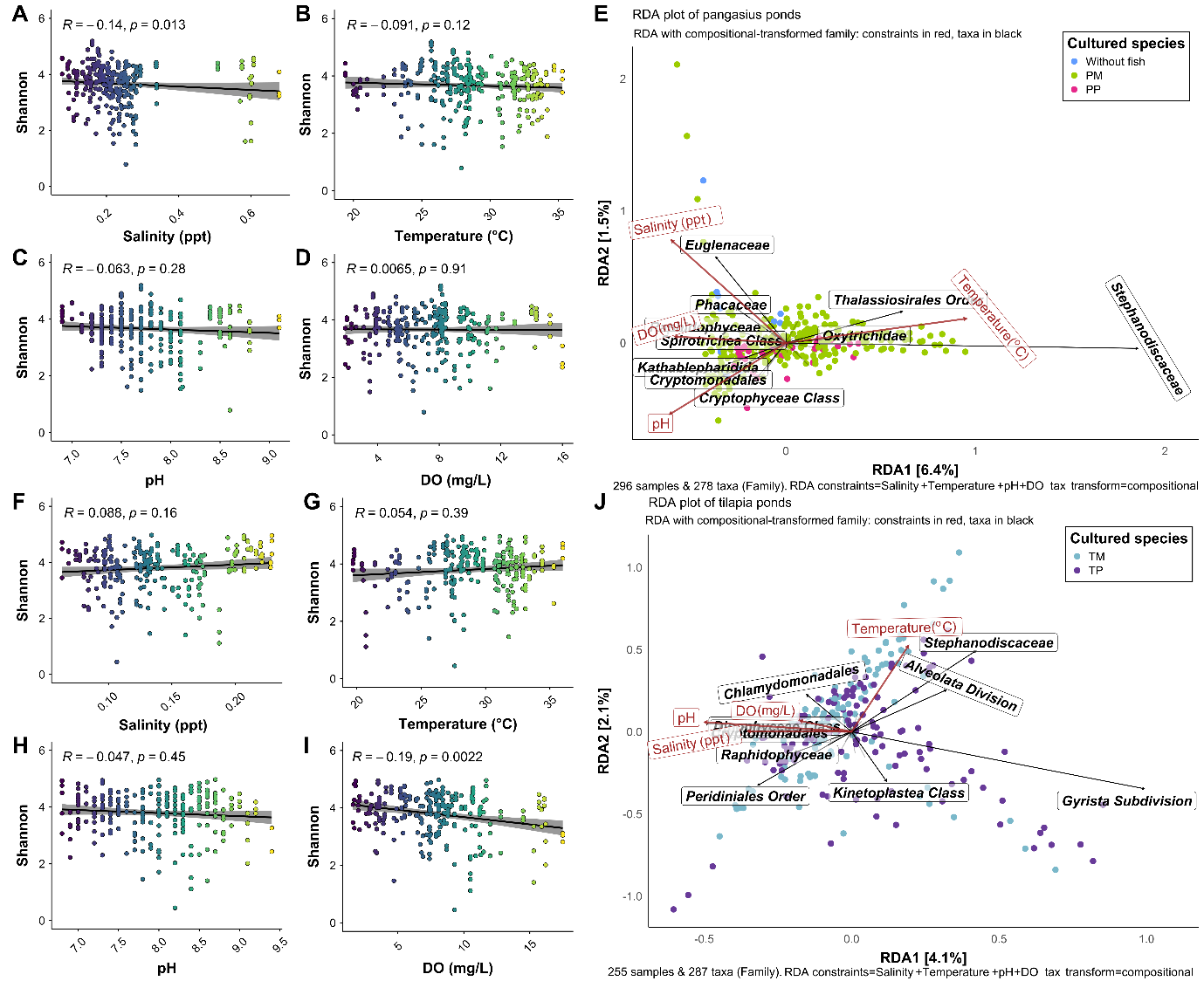


Fig. S3 Spearman’s correlation and redundancy analysis of pangasius and tilapia pond water.

Correlation analyses were carried out between microeukaryotic alpha diversity (Shannon) and salinity, temperature, pH and DO using Spearman’s correlation (linear regression model) for pangasius ponds (A-D) and tilapia ponds (F-I). Redundancy analysis (RDA) showing the correlation among samples (fish species), environmental factors and microeukaryotic families in Pangasius pond (E) and Tilapia pond (J). Black arrows show vectors of the significant microbes at family levels while red arrow shows vectors of the environmental factors. A positive correlation is indicated when the angle between vectors is less than 90°, while a negative correlation is indicated when it is greater than 90°. Any vector perpendicular to each other indicated no correlation.


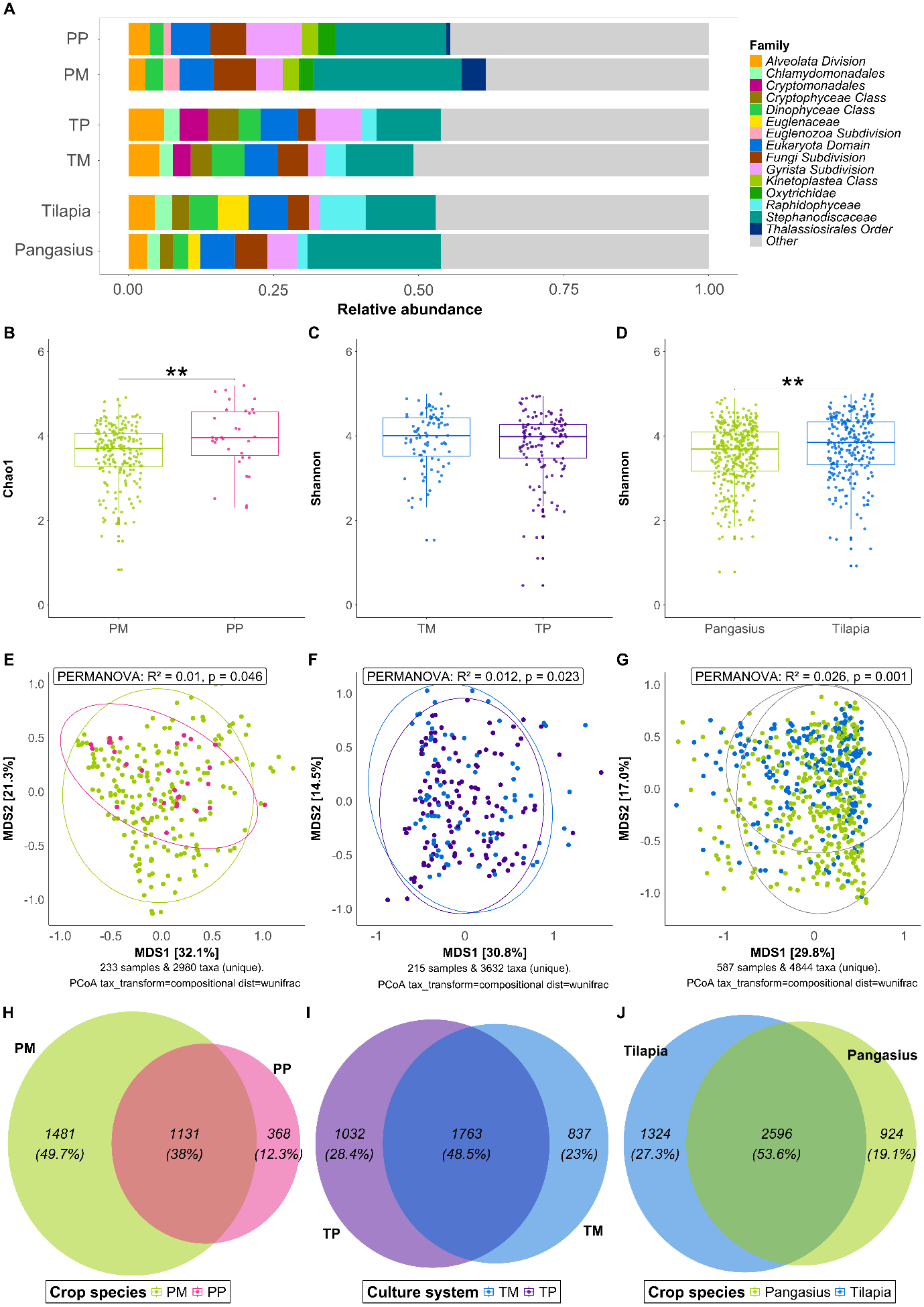


Fig. S4 Microeukaryotic composition, diversity and shared ASVs in different culturing systems.

A) Microeukaryotic composition of top 10 classified and unclassified families in PM (Pangasius-monoculture), PP (Pangasius-polyculture), TM (Tilapia-monoculture), TP (Tilapia-polyculture), pangasius and tilapia (ponds with only pangasius and only tilapia) culture systems. B) Alpha diversity (Shannon) of pangasius-monoculture and polyculture system, C) tilapia-monoculture and polyculture system, D) pangasius and tilapia ponds. E) PCoA ordination with weighted-UniFrac distance matrix between PM and PP systems, F) between TM and TP systems, G) between pangasius and tilapia culture systems. Shared and unique ASVs between PM and PP (H), TM and TP (I), and pangasius-tilapia (J). Asterisks indicate statistically significant differences between groups using the Kruskal-Wallis test (*: *p*-value < 0.05; **: *p*-value < 0.01; ***: *p*-value < 0.001; ****: *p*-value < 0.0001).


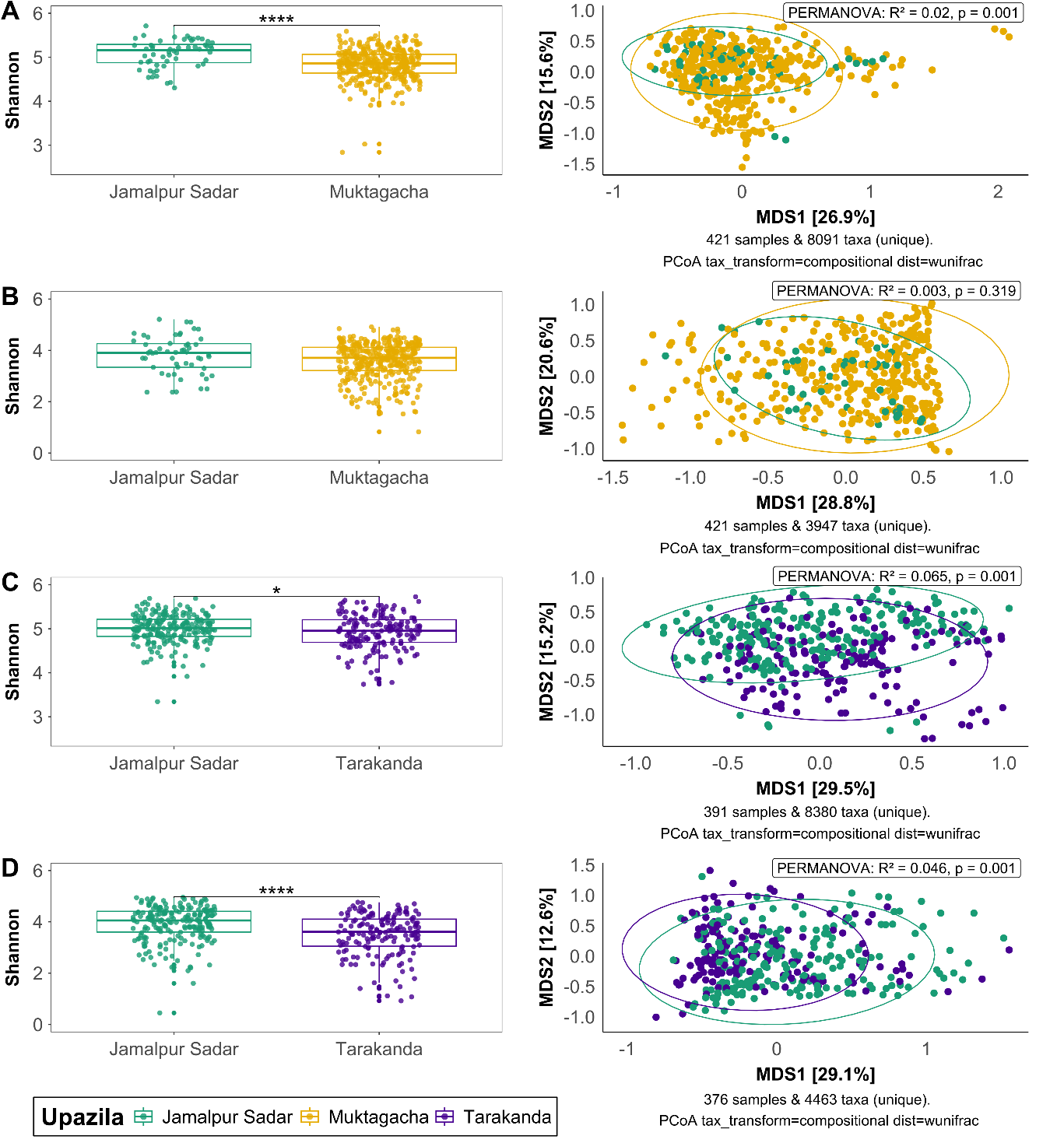


Fig. S5 Microbial diversity in pangasius and tilapia culture ponds across different geographical locations.

Shannon diversity (left), beta diversity (PCoA) with weighted-UniFrac distance (right) of bacterial (A) and microeukaryotic (B) communities in pangasius pond water from different upazila. Shannon diversity (left), beta diversity (PCoA) with weighted-UniFrac distance (right) of bacterial (C) and microeukaryotic (D) communities in tilapia pond water from different upazila. Asterisks indicate statistically significant differences between groups with the Kruskal-Wallis test (*: *p*-value < 0.05; **: *p*-value < 0.01; ***: *p*-value < 0.001; ****: *p*-value < 0.0001).
